# Silk Fibroin–Polyphenol Gels and Hydrogels: Molecular Interactions, Gelation Strategies, Responsive Behaviors, and Multifunctional Applications

**DOI:** 10.3390/gels12050436

**Published:** 2026-05-15

**Authors:** Simeng Ma, Zhuanghong Wang, Honghao Fan, Hai He

**Affiliations:** 1 Key Laboratory of Tropical Translational Medicine of the Ministry of Education, School of Public Health, Hainan Academy of Medical Sciences, Hainan Medical University, Haikou 571199, China; 2 NJUST-YX Artificial Intelligence Biomedical Technology Innovation Center, Nanjing University of Science and Technology, Nanjing 210094, China; 3 International Collaborative Research Center for the Development and Utilization of Tropical Food for Special Medical Purpose, School of Public Health, Hainan Academy of Medical Sciences, Hainan Medical University, Haikou 571199, China

**Keywords:** silk fibroin, polyphenols, gels, hydrogels, gelation, stimuli-responsive materials, bioadhesives

## Abstract

Silk fibroin (SF)–polyphenol systems have emerged as a versatile class of gels and hydrogels in which supramolecular interactions and dynamic crosslinking regulate network formation, responsiveness, and multifunctional performance. Polyphenols interact with SF through hydrogen bonding, hydrophobic interactions, π–π stacking, metal coordination, and covalent crosslinking, thereby modulating conformational transitions, gelation behavior, structural stability, and interfacial functionality. These interaction mechanisms enable the development of SF–polyphenol gel systems with tunable mechanical properties, wet adhesion, antioxidant activity, self-healing capability, and stimuli responsiveness. This review summarizes recent advances in SF–polyphenol gels and hydrogels, with particular emphasis on molecular interaction mechanisms, gelation and fabrication strategies, responsive behaviors, and structure–property relationships. Representative preparation approaches, including solution blending, electrospinning, impregnation–adsorption, enzymatic crosslinking, metal–phenolic coordination, and photo-initiated processing, are systematically discussed in relation to their effects on network architecture and functional output. The responsive behaviors of these systems under pH, redox, electrical, thermal, and optical stimuli are also analyzed from the perspective of dynamic gel networks and adaptive material design. Emerging applications of SF–polyphenol gels in bioadhesives, delivery platforms, flexible bioelectronics, wound-related materials, and sustainable functional systems are highlighted. Current limitations associated with polyphenol instability, formulation sensitivity, reproducibility, and scale-up are critically discussed, together with future opportunities for predictive design of gel-based natural polymer systems. This review provides a comprehensive framework for understanding SF–polyphenol gelation and for guiding the development of next-generation multifunctional gels and hydrogels.

## 1. Introduction

Silk fibroin (SF) is a natural structural protein composed of hydrophobic heavy chains (H-chains, 325–350 kDa), hydrophilic light chains (L-chains, 25–26 kDa), and a P25 glycoprotein (25 kDa) through a single disulfide bond between Cys-20 of the L-chain and Cys-172 of the H-chain, as well as through hydrophobic interactions. The P25 glycoprotein associates non-covalently with the H-L heterodimer at a 6:6:1 molar ratio, further stabilizing the macromolecular conformation and participating in the regulation of secretion and folding [1,2,3,4,5]. The H-chains form rigid β-sheet nanocrystals through highly repetitive Gly-X sequences (where X is mainly Ala, Ser, and Tyr), thereby conferring structural stability. By contrast, the amorphous regions are composed of disordered sequences such as Gly-Ala-Gly-Ala-Gly-Y, where the L-chains and P25 glycoprotein are predominantly distributed, jointly regulating flexibility and solubility [6]. The synergistic interaction between these domains gives SF a hierarchical nanophase structure consisting of alternating crystalline and amorphous regions [1]. This unique architecture endows SF with tunable conformations, good mechanical performance, and versatile processability into films, hydrogels, nanoparticles, fibers, and coatings, making it an attractive platform for functional material design [7]. However, pristine SF still exhibits limited intrinsic functionality, particularly in terms of interfacial activity, antioxidant performance, and adaptive responsiveness in aqueous or complex environments. Therefore, molecular functionalization is often required to broaden its applicability in advanced natural polymer systems.

### 1.1. Polyphenols as Functional Regulators in Gel Systems

Polyphenols are a ubiquitous class of plant-derived secondary metabolites, abundant in sources such as tea, grape seeds, and legumes [8]. Owing to their aromatic structures and abundant phenolic hydroxyl groups, these compounds are attractive modifiers for natural polymer materials, as they can provide antioxidant, antibacterial, adhesive, and crosslinking properties [9]. At the same time, the intrinsic instability of polyphenols under environmental stimuli, together with their limited solubility and variable retention stability, can limit their direct use in material systems [10]. Consequently, integrating polyphenols with suitable polymer matrices has become an effective strategy to improve their stability, regulate their interactions, and translate their molecular functions into useful material properties.

### 1.2. SF as a Model Matrix for Polyphenol-Mediated Assembly

**Figure 1:**
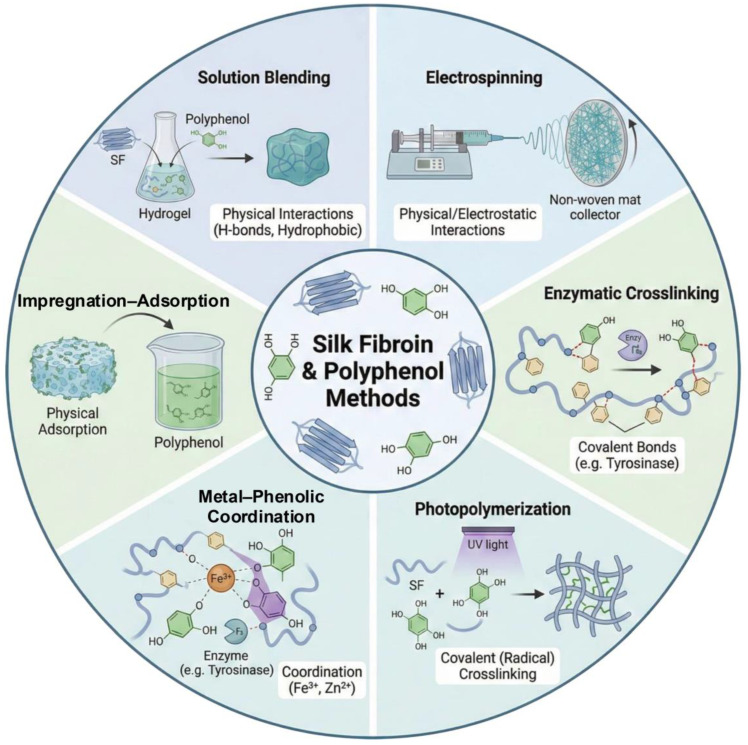
Diverse fabrication strategies leverage specific molecular interactions to create SF–polyphenol composite materials with tailored structures and properties for a range of functional applications.

In the fabrication of functional delivery systems, the specific molecular interactions between polyphenols and their carrier matrices dictate critical performance metrics, including encapsulation efficiency, interfacial stability, and release kinetics. Polyphenols form complex assemblies with biological macromolecules or synthetic polymers, driven by a diverse repertoire of interactions, including hydrogen bonding, hydrophobic effects, π–π stacking, electrostatic forces, van der Waals forces, and metal coordination. Among biological macromolecules, polysaccharides such as cellulose, chitosan, starch, alginate, and pectin constitute another major class of natural polymer matrices in which polyphenol-mediated interactions play similarly important roles. In these systems, hydrogen bonding, hydrophobic association, and covalent or coordination-based crosslinking also govern matrix organization, stability, and functionality. Unlike polysaccharide-based matrices such as alginate and agarose, SF offers a proteinaceous and structurally programmable matrix for polyphenol-mediated assembly. Although alginate hydrogels are widely used because of their mild ionic gelation and cytocompatibility, native alginate lacks intrinsic mammalian cell-adhesive motifs and often requires peptide, gelatin, or extracellular-matrix modification to improve cell–matrix interactions; moreover, ionically crosslinked alginate networks may undergo mechanical weakening or partial disintegration in physiological media because of ion exchange and the dynamic nature of ionic crosslinks [11]. Agarose hydrogels are thermally reversible and structurally stable, but native agarose is generally bioinert and lacks specific cell-adhesive motifs, which limits its use as an actively remodeling bioactive matrix [12]. In contrast, SF combines tunable β-sheet crystallization, adjustable molecular weight, controllable degradation, and hierarchical assembly, providing a more interactive protein platform for constructing bioactive polyphenol-containing gels. The specific nature, intensity, and synergistic interplay of these intermolecular forces critically govern the supramolecular architecture and physicochemical properties of the resulting composite systems [12]. Hydrogen bonding denotes the electrostatic attraction formed between the electropositive end of a donor A–H dipole and the electronegative end of an acceptor B dipole. Polyphenols act as hydrogen donors, forming hydrogen bonds with polar groups on the carrier to enhance complex stability, regulate solubility, and modulate antioxidant activity. At the SF–polyphenol interface, a hydrogen-bonding network is established between the abundant phenolic hydroxyl groups of polyphenols and the amide groups of the SF peptide backbone, including carbonyl oxygen atoms (C=O) and amide N–H protons, as well as polar side-chain residues such as serine and tyrosine. Phenolic hydroxyl groups can act as hydrogen-bond donors, forming O–H···O=C interactions with the amide carbonyl groups in the silk fibroin backbone. Conversely, the oxygen atoms of phenolic hydroxyl groups can serve as hydrogen-bond acceptors by accepting protons from backbone amide N–H groups to form N–H···O–Ar hydrogen bonds, or from O–H groups on polar side chains such as serine and tyrosine to form O–H···O–Ar interactions. Polyphenols enriched with pyrogallol or galloyl motifs, such as tannic acid, epigallocatechin gallate (EGCG), and gallic acid derivatives, contain multiple phenolic hydroxyl groups that can form multivalent hydrogen-bonding crosslinks with SF segments. These interfacial interactions enhance the mechanical strength, thermal stability, and controlled-release performance of the resulting composites while preserving the intrinsic antioxidant and antibacterial bioactivities of the polyphenols. Hydrophobic interactions further drive assembly by embedding aromatic rings into the hydrophobic domains of the SF through interfacial water release. π–π stacking, generated by the overlap of π-electron clouds between aromatic rings, acts as a key cooperative force. Along with hydrogen bonds and hydrophobic interactions, it provides the stability required for the assembly of polyphenol-biopolymer complexes [13]. Electrostatic attraction occurs between deprotonated phenolic hydroxyls (anions) and protonated amino groups (cations) in near-neutral or slightly alkaline environments. This force, sensitive to pH and ionic strength, enables reversible control of complex formation, thereby influencing solubility, stability, and bioactivity [14]. Van der Waals forces are ubiquitous, dipole-driven intermolecular interactions that are weaker than hydrogen bonds. Yet, despite their lower magnitude, they critically complement hydrogen bonding in polyphenol–protein systems. They reinforce interfacial binding, thereby fine-tuning key physicochemical attributes such as protein conformational stability and rheological characteristics [15,16] (Figure 1).

### 1.3. Scope and Organization of This Review

Leveraging the processable hierarchical architecture of SF and the multi-functional binding capabilities of polyphenols, their complexes have been successfully developed into diverse materials, including hydrogels, nanoparticles, 3D-printable inks, sealants, and flexible bioelectronics. However, despite this broad application potential, current research remains largely confined to macroscopic phenomenological characterization, lacking a systematic understanding in critical areas such as the quantitative structure-activity relationships (QSAR) linking polyphenol chemistry to SF assembly kinetics, long-term stability, and release profiles in complex physiological environments, and the development of standardized, scalable processing routes for clinical and industrial translation. This review addresses these gaps by focusing on the molecular interaction mechanisms and rational design principles of SF–polyphenol complexes, while also highlighting their conceptual parallels with polysaccharide-based systems. In this sense, SF–polyphenol assemblies are discussed not only as a specific material platform, but also as a representative natural-polymer model for understanding polyphenol-mediated structure–property regulation. We systematically summarize fabrication strategies, advanced characterization techniques, and performance modulation approaches, highlighting recent breakthroughs in biomedical engineering, sustainable food packaging, and flexible electronics. The review is comprehensively organized to cover assembly paradigms and fabrication pathways, structure–property relationships and key governing factors, diverse application landscapes, and critical challenges with future perspectives. To place SF–polyphenol systems within a broader natural polymer context, selected comparisons with representative polysaccharide-based gel systems are also briefly discussed.

This review emphasizes not only reported preparation routes and applications, but also the critical structure–property rules, benchmarkable figures of merit, and unresolved translation barriers that determine whether SF–polyphenol gels can move from proof-of-concept demonstrations to reproducible materials platforms.

## 2. Molecular Interaction Mechanisms Governing SF–Polyphenol Gel Formation

### 2.1. Non-Covalent Interactions in SF–Polyphenol Gels

SF–polyphenol interactions involve supramolecular self-assembly driven by structural complementarity and non-covalent interactions. Polyphenols contribute through their phenolic hydroxyls and aromatic rings, while SF’s amide groups and hydrophobic domains confer conformational plasticity, enabling a dynamic transition from random coils to β-sheet structures upon binding. Metal coordination or covalent crosslinking can further strengthen the composite network and enhance its stability [17,18]. The coupling of these components facilitates effective molecular-scale anchoring, which propagates to the mesoscopic level, forming stable network-like or particulate structures. This multi-scale structural integration directly leads to synergistic improvements in macroscopic properties, thereby optimizing the material’s mechanical performance, adhesiveness, drug-delivery capabilities, and electroactivity [19,20,21,22,23,24,25].

Hydrogen bonding is a dominant driving force in the assembly of SF–polyphenol complexes [26]. Polyphenols’ phenolic hydroxyl groups form a high-density hydrogen-bonding network with SF’s amide moieties (–C=O, –N–H) and polar side-chain residues. This interaction modulates SF’s secondary structure, triggering a conformational transition from random coils to β-sheet-enriched domains, thereby bolstering the composite’s thermodynamic stability and mechanical robustness [26,27]. Beyond hydrogen bonding, hydrophobic interactions drive the aggregation of polyphenol aromatic rings with SF’s hydrophobic peptide segments. This entropically driven process excludes interfacial water, thereby densifying the complex architecture. Furthermore, π–π stacking interactions, occurring between polyphenols or with SF’s aromatic residues, such as tyrosine, impose directional constraints, thereby enhancing supramolecular ordering [17,18]. π–π stacking interactions generally adopt three representative geometries on the potential energy surface: parallel-displaced (PD), in which one aromatic ring is laterally offset above another; T-shaped (T), in which one ring is oriented approximately perpendicular to the plane of the other; and sandwich-like (S), in which two aromatic rings are nearly cofacial and directly superimposed [28]. Parallel-displaced stacking between the galloyl groups of polyphenols and the phenyl rings of tyrosine residues in SF can stabilize transient interchain contacts, restrict side-chain fluctuations, and facilitate the formation of β-sheet structures. T-shaped stacking may also serve as a transient interaction hub that suppresses side-chain mobility and spatially coordinates the formation of interchain hydrogen bonds [29]. In hydrated gel environments, hydrogen bonds are highly dynamic and reversible because of competition with surrounding water molecules, whereas π–π stacking can provide additional enthalpic stabilization and promote more ordered supramolecular alignment between polymer chains [30]. In near-neutral or alkaline environments, the partial deprotonation of phenolic hydroxyls introduces electrostatic interactions, making complexation highly pH- and ionic-strength-dependent. Complementing these, Van der Waals interactions synergistically strengthen the molecular interface and modulate the microscopic organization of the resulting gels or networks [31]. These interactions act collectively, driving a stepwise structural evolution from initial anchoring and conformational switching to physical aggregation and densification, ultimately yielding a composite system with a dynamically organized hierarchical structure [20,32,33,34,35,36,37,38].

Beyond non-covalent interactions, stable cross-linking fortifies SF–polyphenol complexes. Enzymes, alkaline pH, or free radicals oxidize polyphenols into reactive quinone intermediates. These subsequently conjugate to SF’s nucleophilic residues via Michael addition or Schiff base reactions, forming a dense covalent network that significantly improves structural stability, swelling resistance, and proteolytic stability [39]. On the other hand, the ortho-phenolic hydroxyl groups facilitate dynamic coordination to metal ions (e.g., Fe^3+^, Cu^2+^, Zn^2+^). This generates a robust yet reversible metal–polyphenol network (MPN) that intertwines with the SF hydrogen-bonded matrix, endowing the complexes with self-healing, wet adhesion, and stimulus responsiveness [20,40,41]. The densified SF matrix synergistically shields polyphenols from enzymatic degradation, enabling sustained release, while polyphenols concurrently modulate SF conformational transitions and confer intrinsic bioactivities, such as antioxidant and antibacterial effects. This mutual reinforcement enhances the material’s potential for tissue engineering, drug delivery, and food preservation [13,15].

### 2.2. Structure–Activity and Structure–Property Relationships

**Figure 2:**
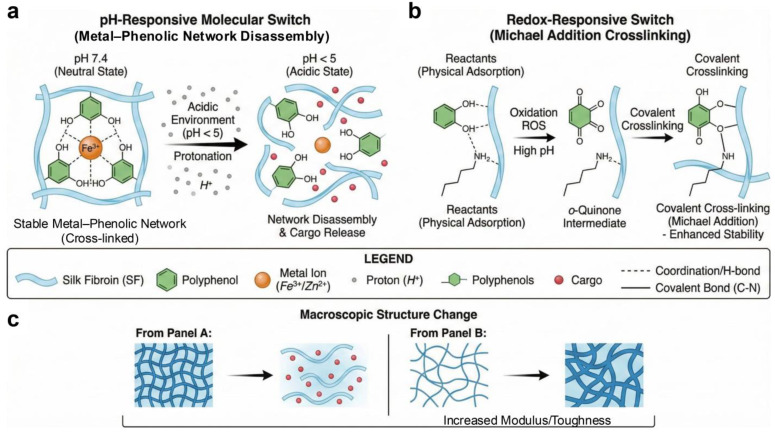
Schematic illustration of molecular switching mechanisms in stimuli-responsive SF–polyphenol complexes. (**a**) pH-responsive disassembly of metal–phenolic networks for cargo release. (**b**) Redox-induced covalent crosslinking via quinone-mediated Michael addition. (**c**) Macroscopic outcomes of network disassembly and reinforcement.

QSARs in SF–polyphenol complexes reveal that the efficiency of SF structural transformation is critically governed by polyphenol molecular architecture, specifically the density of phenolic hydroxyl groups and molecular weight. Polyphenols rich in galloyl moieties, such as tannic acid (TA), act as multivalent crosslinkers that can engage multiple hydrogen-bonding sites on adjacent SF chains, either simultaneously or sequentially. The clustered galloyl groups provide dense arrays of phenolic hydroxyl donors and aromatic surfaces, enabling cooperative, multivalent binding with backbone amide carbonyls, amide N–H groups, and polar side chains of SF. Once the first galloyl–SF contact is established, the local concentration of neighboring binding sites increases, thereby reducing the entropic penalty for subsequent interactions and promoting progressive interchain association. This cooperative binding process restricts chain mobility, dehydrates the local protein environment, and brings hydrophobic Gly–Ala-rich segments into closer proximity, which accelerates the conformational transition of SF from random coil or α-helix structures toward β-sheet-rich domains (Table 1) [42]. The molecular weight strongly influences polyphenol–silk fibroin interactions: high-molecular-weight polyphenols drive rapid gelation through extensive inter-chain bridging, whereas smaller phenolics primarily bind hydrophobic pockets without inducing immediate macroscopic phase separation. The rigid aromatic backbone of polyphenols further imposes steric constraints that conformationally lock and stabilize the SF network. This structure–function relationship is clearly evidenced in SF–TA complexes, where the amide I band shifts from 1640 cm^–1^ to 1623 cm^–1^ in a TA concentration-dependent manner, directly reflecting progressive induction of β-sheet crystallinity [43] (Figure 2).

### 2.3. Characterization and Structure–Property Relationships of SF–Polyphenol Gels

SF combines excellent biocompatibility with tunable mechanics, derived from the induced crystallization of its repetitive peptide motifs into stable β-sheets. Polyphenols, characterized by their functional hydroxyl groups and hydrophobic aromatic rings, contribute diverse bioactivities, including UV shielding and antioxidant performance. The combination of these two components yields a material system governed by a mechanism of intermolecular interaction-driven structural reconstruction. Polyphenols efficiently govern the conformational changes and network structure of SF through a wide range of interactions, from hydrogen bonding and π–π stacking to metal coordination and oxidative cross-linking. Consequently, this microstructural tuning results in significant variations in macroscopic properties, enabling precise tailoring of mechanical robustness, degradation rates, and biological responses (Table 2). Although the representative examples summarized here mainly focus on SF-based systems, similar interaction principles and functional outcomes have also been reported in polysaccharide-based matrices.

#### 2.3.1. Spectroscopic and Structural Characterization

To comprehensively elucidate the multi-scale structure of SF–polyphenol complexes, a diverse array of characterization techniques is employed. Molecular interactions and conformational transitions are typically probed via spectroscopic methods, including Fourier transform infrared spectroscopy (FTIR), circular dichroism (CD), UV–visible spectroscopy (UV–Vis), and fluorescence spectroscopy. The crystalline structure and thermal properties are analyzed using X-ray diffraction (XRD) and differential scanning calorimetry (DSC), respectively. Additionally, morphological characteristics and particle dynamics are observed through high-resolution microscopy techniques, including scanning electron microscopy (SEM), transmission electron microscopy (TEM), and atomic force microscopy (AFM), as well as through dynamic light scattering (DLS).

A comprehensive multi-scale characterization strategy is employed to elucidate the structural evolution of SF–polyphenol complexes. At the molecular level, FTIR and CD spectroscopies provide critical insights into secondary-structure transitions. For instance, in SF–TA complexes, the redshift of the amide I band (1640 cm^–1^, 1623 cm^–1^) and the emergence of a characteristic β-sheet peak at 1533 cm^–1^ in FTIR indicate a polyphenol-induced conformational transition from random coils to stable β-sheet crystals [43]. In addition to the amide I and II regions, the amide III region can provide complementary evidence for SF backbone rearrangement because it is mainly associated with C–N stretching and N–H bending vibrations. In silk fibroin, the amide III band has been reported around 1230–1260 cm^−1^, with the lower-wavenumber component near 1230 cm^−1^ being related to coil-rich structures and the higher-wavenumber region around 1260 cm^−1^ being associated with silk II/β-sheet features [61,62]. In SF–polyphenol-related systems, direct evidence of a distinct amide III shift remains limited; nevertheless, a gallic acid–Fe coordination network deposited on silk fibroin microspheres shifted the band from 1230 to 1250 cm^−1^, accompanied by a shoulder at 1325 cm^−1^ assigned to phenolate C–O–Fe vibration, suggesting that polyphenol-derived coordination/hydrogen-bonding networks can perturb the local chemical environment of the SF backbone [63]. Similarly, in a TA-containing coassembly of multi-surface-charged chitin nanofibers (OAChN)–TA–SF adhesive system, the amide III band of SF was observed at 1239 cm^−1^, together with amide I and II bands at 1658 and 1516 cm^−1^, while the O–H/N–H stretching band shifted to approximately 3200 cm^−1^, indicating extensive intermolecular hydrogen bonding among TA, SF, and OAChN [64]. However, some SF–TA or polyphenol-loaded SF systems report an amide III band around 1228–1235 cm^−1^ without explicitly demonstrating a clear peak shift, indicating that amide III changes are system-dependent and should be interpreted together with amide I/II shifts, β-sheet-associated bands, and CD results [19,26,65]. This ordering is corroborated by CD spectra, which show a pronounced positive ellipticity at 195 nm and a distinct negative ellipticity at 212 nm, confirming the rearrangement of molecular chains into highly ordered architectures [17,20]. This molecular ordering results in enhanced crystallinity, as evidenced by XRD patterns showing a peak shift from 20° to 24° and a significant increase in the crystalline fraction [64]. Consequently, DSC analysis reveals that strengthened intermolecular hydrogen bonds elevate the thermal decomposition temperature, indicating improved thermal stability [37].

Morphologically, electron microscopy (SEM/TEM) unveils the hierarchical assembly of these complexes. TEM observations of SF–TA hydrogels disclose a network formed by the interweaving of approximately 7 nm SF and 5 nm TA nano-bundles [47]. Similarly, SEM imaging of electropunk SF/Gel/GA membranes reveals a uniform, continuous nanofiber network (diameter: 134 nm) [66]. While SF/TA/Fe^3+^ cryogens exhibit a honeycomb-like porosity where pore size inversely correlates with compressive modulus [67]. Furthermore, for particulate systems, DLS is used to monitor size distributions; for example, SF–proanthocyanidin cross-linked nanoparticles and Rosmarinus acid-loaded SFNs exhibited average hydrodynamic diameters of 120.1 nm and approximately 255 nm, respectively [19,68].

#### 2.3.2. Mechanical Properties

SF is renowned for its exceptional mechanical robustness, underpinned by its unique amphiphilic molecular architecture [69]. The alternating hydrophilic and hydrophobic motifs drive the self-assembly of SF into stable antiparallel β-sheet nanocrystals. These crystalline domains function as physical cross-links, interconnected by dense hydrogen-bonding networks, endowing the material with stiffness and tensile strength that surpass those of many natural counterparts, such as collagen, chitosan, and alginate, and even rival those of weight-bearing biological tissues [70]. In SF–polyphenol complexes, this mechanical baseline is further elevated through a synergistic reinforcement strategy. Polyphenols [e.g., TA, gallic acid (GA), EGCG] establish a dense repertoire of non-covalent interactions, including hydrogen bonds, hydrophobic effects, and π–π stacking, with the SF backbone. This multipoint anchoring accelerates gelation and imparts superior adhesion and shear recovery; for instance, SF–TA hydrogels exhibit a 10-fold increase in compressive strength relative to pure SF [71]. Furthermore, the introduction of metal ions enables a secondary enhancement via metal–phenolic coordination. In ternary systems (e.g., m-SF/TA/Zn^2+^), a dual network forms in which polyphenols bridge SF chains via hydrogen bonds and coordinate to Zn^2+^ ions. This cooperative mechanism is essential for gelation; rheological analyses indicate that the ternary system exhibits a significant sol–gel transition (where the G’ exceeds the G’’), whereas the binary SG–TA (m-SF/TA) system, which depends solely on hydrogen bonding, persists in a solution state [55].

#### 2.3.3. Functional Properties

SF, distinguished by its structural robustness and biocompatibility, serves as an ideal biopolymeric carrier for stabilizing and delivering polyphenolic compounds. The assembly of SF–polyphenol complexes is driven by dense hydrogen bonding and hydrophobic interactions [42,72]. Relevant research highlights this enhanced stability, showing that SF-encapsulated rosemary extract retained approximately 85% of its antioxidant activity (DPPH scavenging) after 24 h of incubation at 37 °C, demonstrating superior performance compared to free extracts. Furthermore, these SF-based nanocarriers proved effective at protecting various plant-derived polyphenols against harsh conditions, maintaining bioactivity levels above 80% even under exposure to 70 °C and light irradiation [73].

#### 2.3.4. Stability, Biodegradability, and Safety Considerations

SF, renowned for its intrinsic biocompatibility and biodegradability, serves as a versatile platform for biomedical applications. The strategic incorporation of polyphenols (i.e., TA, GA, catechins) endows the SF matrix with potent antioxidant, anti-inflammatory, and antibacterial properties [14]. Supramolecular assembly via hydrogen bonding, hydrophobic effects, and π–π stacking improves surface wettability and cell anchorage. In vitro and in vivo studies demonstrate high biosafety, with SF–polyphenol complexes showing minimal cytotoxicity and maintaining 80–90% viability in fibroblasts, keratinocytes, endothelial cells, and stem cells [18,21,27]. Notably, the SF–TA hydrogels developed by Gao et al. [18]. The network formation was attributed to multiple non-covalent interactions between SF and TA, which were systematically examined using attenuated total reflectance Fourier transform infrared spectroscopy (ATR-FTIR), DSC, solid-state carbon-13 cross-polarization magic-angle spinning nuclear magnetic resonance (^13^C CP-MAS NMR), pH-dependent experiments, urea dissociation tests, and rheological measurements. FTIR peak shifts and urea-induced dissociation provided evidence for the participation of hydrogen bonding in network stabilization, while DSC and solid-state NMR suggested that TA restricted SF chain mobility and altered its secondary structure. These findings support a synergistic assembly model in which initial hydrophobic association is followed by hydrogen-bond-mediated stabilization, potentially accounting for the strong cohesion, wet tissue adhesion, and aqueous stability of TASK. Nevertheless, these findings primarily establish the presence of SF–TA interactions and their association with macroscopic performance, but they do not fully resolve the relative contributions of hydrogen bonding, hydrophobic association, chain entanglement, and SF conformational changes to the observed mechanical, adhesive, and biological properties. Further studies integrating in situ rheology, competitive disruption of specific interactions, interfacial adhesion energy analysis, and quantitative structure–property correlations are therefore needed to validate the proposed mechanism more rigorously. Similarly, bacterial cellulose-based dressings functionalized with SF and EGCG have been developed to improve cell–material interactions and promote infected wound healing [23]. SF–polyphenol modification can therefore extend beyond hydrogel network reinforcement and contribute to wound microenvironment regulation through the combined effects of cytocompatibility, antibacterial activity, and pro-healing bioactivity. This strategy has also been expanded to other tissue-repair scenarios. For example, CS/TA/SF supramolecular hydrogels can serve as wet-adhesive hemostatic sealants, reducing blood loss and shortening hemostasis time in arterial and visceral bleeding models [74]. Meanwhile, STIG hydrogels composed of SF, TA, ibuprofen, and guanidine hydrochloride (GuCl) integrate moisture-responsive adhesion with localized anti-inflammatory drug delivery, enabling annular fissure sealing and alleviating inflammation-associated intervertebral disc degeneration after microdiscectomy [75]. These studies highlight the multifunctionality of SF–polyphenol-based systems in modulating diverse pathological microenvironments, including infected wounds, hemorrhagic tissues, and inflamed intervertebral discs. However, these biological outcomes should be interpreted as integrated system-level responses rather than direct evidence for a single dominant mechanism, as wound repair, hemostasis, adhesion, and disc protection may be jointly influenced by matrix structure, interfacial interactions, degradation behavior, drug release, antibacterial activity, and host inflammatory responses.

Collectively, SF–polyphenol composite systems have shown favorable biocompatibility and preliminary biosafety, which provides an important basis for their potential clinical translation. For example, the chitosan/SF/TA–Fe^3+^ photothermally responsive cryogel maintained good cell compatibility after repeated photothermal cycles and caused negligible inflammation or tissue necrosis in vivo [76]. SF-based or polyphenol-containing hydrogel systems have been evaluated via histopathological analyses of major organs, such as the liver and kidneys, thereby supporting their short-term biosafety within appropriate therapeutic windows [50]. Nevertheless, these findings should be interpreted with caution, as most available biosafety evidence still derives from short-term cytocompatibility assays, limited animal models, and conventional histological observations. Potential risks, including dose-dependent cytotoxicity, chronic inflammation, metabolic burden, and long-term tissue accumulation, remain insufficiently resolved. For instance, high TA concentrations may partially suppress cell proliferation, whereas lower TA contents commonly used in composite formulations generally exhibit better cytocompatibility [77,78]. In addition, hydrolyzable tannins can be enzymatically or microbially degraded into smaller phenolic metabolites, such as gallic acid and pyrogallol, and TA can be hydrolyzed into gallic acid and glucose components, suggesting the possibility of metabolic transformation and clearance [79,80,81]. However, this should not be taken as evidence that all SF–polyphenol materials are intrinsically risk-free. Rather, their biosafety should be regarded as a system-dependent outcome governed by material composition, crosslinking density, degradation behavior, release kinetics, administration dose, and application site. Therefore, long-term in vivo degradation, metabolic clearance, immune response, hemocompatibility, organ toxicity, and dose–response relationships should be systematically investigated to more rigorously evaluate their translational potential.

Despite favorable in vivo outcomes, several critical hurdles remain for the clinical translation of SF–polyphenol complexes, primarily concerning toxicity and processing challenges. A delicate balance must be maintained between material performance and biosafety, as high concentrations of TA (>10 wt%) can dramatically enhance mechanical modulus and adhesion but have been shown to marginally inhibit cell proliferation, necessitating careful optimization of the polyphenol-to-protein ratio to maximize crosslinking density without exceeding the metabolic threshold of local tissues. For systems utilizing MPNs, the long-term fate of transition metal ions (e.g., Fe^3+^, Cu^2+^) requires rigorous assessment, as local accumulation from degrading scaffolds could induce oxidative stress or tissue discoloration despite their essential roles [42,82]. Furthermore, sterilization compatibility presents a critical yet often overlooked challenge: standard autoclaving can induce uncontrolled β-sheet crystallization or protein denaturation in SF, while gamma irradiation may degrade the polyphenol structure, making the development of non-destructive sterilization protocols (e.g., ethylene oxide or supercritical CO_2_) that preserve the integrity of the supramolecular network an essential prerequisite for industrial production and regulatory approval [43,83]. Previous studies have shown that autoclaving can increase the stiffness and β-sheet content of SF scaffolds and reduce their degradation rate, whereas γ-irradiation may accelerate degradation or induce dose-dependent molecular weight reduction [84,85]. Ethylene oxide (EtO) is more suitable for heat- and moisture-sensitive SF–polyphenol networks, but sufficient aeration or post-sterilization leaching is required to minimize residual toxicity [86]. In SF nanoparticle suspensions, ≥5 kGy was sufficient to achieve effective sterilization, whereas <5 kGy failed to eliminate aerobic and facultative anaerobic microorganisms [87]. Supercritical CO_2_ sterilization also represents a promising low-temperature option for sensitive biomaterials, particularly when combined with mild additives [88]. Therefore, post-sterilization verification of β-sheet content, FTIR/CD spectra, molecular weight, rheological behavior, adhesive strength, degradation profile, residual toxicity, and cytocompatibility should be required before industrial translation.

The ability to degrade and be metabolized by the host is a distinct advantage of SF-based implants, ensuring long-term biosafety without the need for removal [7,27]. SF–polyphenol complexes, degradation kinetics are intrinsically governed by the molecular architecture. Polyphenols (e.g., TA, EGCG) establish a dynamic supramolecular network with SF via dense hydrogen bonding and hydrophobic interactions. The conformational packing of SF chains is modulated by these interactions, which, in turn, dictate the materials’ structural stability and susceptibility to proteolytic degradation [17,20,23]. While pure SF degrades relatively slowly, the composite system exhibits remarkable tunability. A bio-adhesive composed of SF, TA, and soybean meal exhibited a mass loss of approximately 46.45% over 30 days of soil burial, underscoring its potential for environmental degradation [37]. Similarly, 3D-printed SF–polyphenol complexes have been validated in animal models for gradual resorption post-surgery [27]. Mechanistically, the degradation is primarily driven by enzyme-mediated proteolysis. In vitro assays with Protease XIV typically reveal a linear mass-loss profile, reflecting the accelerated breakdown of the protein matrix [66]. Physiological fluid erosion and cell-mediated resorption exacerbate this process in vivo, as evidenced by the slow breakdown of nano-chitin/SF/TA adhesives during tissue healing [64]. The degradation rate of SF–polyphenol complexes is primarily determined by crosslinking density and microarchitecture, thereby enabling precise tuning via polyphenol loading, supplementary crosslinking strategies, or pore optimization [89]. Modifying polyphenol content or adding auxiliary cross-linkers enables customization of the material’s longevity to align with the precise temporal needs of tissue regeneration and long-term safety. Collectively, spectroscopic, microscopic, thermal, and rheological analyses indicate that polyphenols regulate SF gel systems by coupling conformational ordering with hierarchical network densification.

A critical analysis of the literature shows that stronger bioactivity is not always coupled with stronger gels. TA-rich systems usually provide rapid gelation, wet cohesion, and antioxidant activity, but excessive TA can cause heterogeneous coacervation, phenolic oxidation, or radical-quenching during photopolymerization. EGCG-based systems are attractive for ROS-responsive wound environments because they combine antioxidant activity with enzymatically controlled gelation, whereas small phenolic acids should be treated mainly as auxiliary or grafted motifs rather than validated standalone SF gelators. Therefore, Table 3 summarizes key figures of merit to guide polyphenol selection based on gelation kinetics, mechanical performance, wet adhesion, antioxidant/ROS-responsive behavior, and electrical/strain-sensing performance.

## 3. Gelation and Fabrication Strategies for SF–Polyphenol Gels

The major gelation strategies can be broadly categorized into physical assembly, covalent fixation, and coordination-assisted network formation, each involving distinct trade-offs in stability, processability, and multifunctionality. Methods for fabricating SF–polyphenol complexes are broadly classified into physical assembly and chemical crosslinking. Physical assembly exploits non-covalent interactions (e.g., hydrogen bonding, hydrophobic effects, and π–π stacking), enabling mild, straightforward preparation that preserves polyphenol bioactivity with minimal denaturation or functional loss. Conversely, chemical crosslinking through enzyme-mediated oxidation, MPN coordination, or photopolymerization yields covalently stabilized, highly tunable networks exhibiting outstanding long-term structural integrity. These covalent strategies are especially advantageous for applications demanding resilience under high humidity, extended durability, or stringent mechanical performance.

Before the preparation of SF–polyphenol hydrogels, natural silk is typically used as the raw material, and sericin is removed through a degumming process to obtain purified SF fibers. Commonly used degumming systems include alkaline degumming, acid degumming, enzymatic degumming, soap-based degumming, high-temperature/high-pressure water degumming, and surfactant-assisted degumming [92,93]. Different degumming methods vary in their ability to preserve SF’s molecular weight and the viscosity of the resulting solution. Although Na_2_CO_3_-based degumming can be completed in approximately 30 min, it may still induce partial hydrolysis of peptide chains, reducing molecular weight. In contrast, milder degumming approaches, such as ionic-liquid treatment and enzymatic degumming, can better preserve the native molecular weight of SF [93]. Prolonged degumming further reduces the molecular weight of SF and enriches glycine and alanine residues associated with β-sheet formation, thereby modulating the gelation kinetics and mechanical behavior of the resulting hydrogels [94]. Conversely, shorter degumming times can promote β-sheet crystallite formation and increase mechanical stiffness while reducing the degradation rate [95]. Degummed SF fibers are poorly soluble in water and therefore require specific solvent systems to convert them into regenerated SF solutions suitable for subsequent applications. Early dissolution systems primarily relied on strong inorganic acids, which readily induced degradation of SF macromolecules, thereby compromising material performance. Subsequently, inorganic salt systems such as lithium bromide were widely adopted because they caused relatively less degradation of SF molecular chains. In recent years, ternary inorganic salt–ethanol–water solvent systems and highly polar organic solvent systems have increasingly been used for silk fibroin regeneration, providing additional routes to obtain regenerated SF with distinct structural characteristics [96]. During regeneration, ethanol treatment can effectively induce the conformational transition of SF from a predominantly random-coil structure to a β-sheet-rich structure, thereby regulating the content and distribution of β-sheet crystallites [97].

These pretreatment processes yield regenerated silk fibroin solutions with distinct molecular weights and structural characteristics that serve as key precursors for preparing SF–polyphenol hydrogels. The molecular-level differences introduced by degumming, dissolution, and regeneration can be further amplified during the co-assembly of SF and polyphenols. Typically, SF solutions regenerated through lithium bromide (LiBr) dissolution and dialysis are dominated by random-coil conformations, and β-sheet crystallite formation can be induced by ethanol annealing, pH adjustment, or freeze–thaw treatment. Mild degumming or low-damage extraction methods help preserve the high molecular weight and chain integrity of SF, thereby providing a structural basis for the subsequent formation of continuous, physically crosslinked networks [92,93]. During the co-assembly of SF and polyphenols, galloyl-rich polyphenols, such as tannic acid, can form multiple hydrogen bonds with amide and carbonyl groups along SF chains and promote network densification through multivalent crosslinking and π–π interactions, enhancing the mechanical properties of the hydrogels [42]. Although low-molecular-weight SF generated by excessive degumming may accelerate initial nucleation by increasing exposure of hydrophobic residues, the absence of long-range ordered chain segments hinders the formation of regular, stable, physically crosslinked networks, ultimately reducing the mechanical strength of the hydrogels [94,95].

### 3.1. Physical Assembly Strategies

#### 3.1.1. Solution Blending and In Situ Assembly

Solution blending remains the predominant physical assembly method for silk fibroin–polyphenol complexes. Components are mixed directly in aqueous or mild solvents, with complexation driven by hydrogen bonding, hydrophobic interactions, and π–π stacking. β-sheet induction in SF is commonly employed to densify the network. A typical preparation protocol involves mixing an aqueous SF solution (2–15 wt%) with a polyphenol solution (0.7–10 wt%) at an SF-to-polyphenol mass ratio of 1:0.2–1:5, followed by homogenization under continuous stirring at 300–500 rpm. During mixing, the pH should be adjusted based on the pKa values and the oxidation susceptibility of the selected polyphenol. Polymers typically contain multiple ionizable phenolic hydroxyl groups and/or carboxyl groups, and their apparent pKa values depend on the site and are influenced by the solvent environment, ionic strength, and binding state. For example, the reported pKa values of phenolic hydroxyl groups in commercial tannic acid are widely distributed, typically ranging from approximately 6–8.5, and vary with sample composition and measurement method [98]. In general, the pH should be maintained in a weakly acidic to near-neutral range, below or close to the dominant phenolic pKa, so that most phenolic hydroxyl groups remain protonated and retain their hydrogen-bond-donating ability. Conversely, excessively high pH can promote phenolate formation, which may accelerate autoxidation and quinone formation, leading to color darkening and uncontrolled aggregation [99]. Galloyl-rich TA can form stable supramolecular assemblies with neutral polymers through pH-dependent hydrogen bonding, owing to the multivalent interactions provided by its abundant phenolic hydroxyl groups. Although TA-based hydrogen-bonded multilayers may remain stable until pH values as high as 8–9.5, where extensive phenolic ionization can trigger disassembly, maintaining a mildly acidic pH window of 4.0–6.5 is generally more favorable for stable TA–SF complexation and homogeneous network formation [98,100]. For catechin-type polyphenols such as EGCG, a narrower, mildly acidic pH window is often preferable during solution mixing because these compounds are more prone to deprotonation, autoxidation, dimerization, and degradation under neutral-to-mildly alkaline conditions [101]. For small-molecule phenolic acids, such as gallic acid, caffeic acid, and chlorogenic acid, the contribution of carboxyl pKa values to solubility and charge state should also be considered. Partial ionization of the carboxyl group can improve aqueous solubility, whereas excessive deprotonation of phenolic hydroxyl groups may increase the risk of oxidation and coupling reactions (Table 4). Mixing time is concentration-dependent, ranging from 0.5–2 h for dilute systems (e.g., 0.7 wt% TA) to 12–24 h for concentrated systems (e.g., 10 wt% TA). Ultrasonic treatment is limited to the initial dissolution/dispersion step (<1 h) to prevent shear-induced structural degradation or oxidative damage [20,37,102].

#### 3.1.2. Electrospinning

Electrospinning leverages high-voltage electrostatic fields to fabricate SF–polyphenol nanofibrous membranes, exploiting charge interactions to ensure the homogeneous distribution of components within the fiber matrix. The resulting membranes are characterized by high specific surface areas and tunable porosity, rendering them ideal for wound dressings, bio-adhesive interfaces, tissue scaffolds, and substrates for flexible electronics. A standard protocol involves co-dissolving SF and polyphenols in a spinnable carrier polymer in organic solvents to optimize electrospinnability, followed by electric-field-induced stretching to form uniform fibers [36,113]. Furthermore, advanced emulsion electrospinning enables the engineering of core–shell architectures. This structural encapsulation significantly enhances the stability of polyphenols and enables precise regulation of their release kinetics [114]. In summary, electrospinning is a versatile platform for tailoring functional materials with specific pore microstructures [66,115].

#### 3.1.3. Impregnation–Adsorption Strategy

The impregnation–adsorption approach reflects a typical post-functionalization paradigm. In this methodology, polyphenols infiltrate the surface or pore channels of prefabricated SF matrices, including films, scaffolds, or silk fibroin nanoparticles (SFNs), via non-covalent interfacial anchoring. This methodology offers high operational versatility, endowing the substrate with antioxidant, antibacterial, or adhesive functionalities while preserving its intrinsic architecture [19,59,116]. However, given the reversible nature of physical adsorption, the cargo is susceptible to premature leakage or burst release in response to environmental fluctuations (e.g., shifts in pH or ionic strength) [117]. Post-treatments, including SF β-sheet crystallization-induced densification, formation of secondary interaction networks, or covalent crosslinking, are strategically applied to physically or chemically entrap polyphenols within the composite system, thereby reducing their release and improving retention stability [118].

### 3.2. Chemical Cross-Linking Strategies

Enzymatic oxidation, MPNs, and photopolymerization are three covalent and coordination chemistries that are widely used to manufacture strong SF–polyphenol complexes. These techniques promote the formation of stable, densely cross-linked structures, which are critical for improving mechanical performance and maintaining functional bioactivity [25,32].

#### 3.2.1. Enzymatic Covalent Cross-Linking

In enzymatic strategies, catalysts such as tyrosinase or laccase are typically employed to catalyze the oxidative dehydrogenation of polyphenols, converting phenolic hydroxyl groups into highly reactive *o*-quinones or quinones [32]. Following this activation, these electrophilic quinone species undergo facile reaction with the nucleophilic residues (e.g., free amines or tyrosine) of SF via Schiff base or Michael addition pathways. This process establishes robust C–N or C–O covalent linkages, effectively anchoring the polyphenols to the protein backbone [35]. Enzymatic crosslinking requires a controlled buffer system. Careful adjustment of enzyme concentration, reaction duration, and pH enables precise tuning of crosslinking density while minimizing excessive oxidation that could compromise the integrity of the SF matrix [119].

#### 3.2.2. Metal–Phenolic Coordination Cross-Linking

Metal–phenolic coordination exploits the strong chelation between the catechol or galloyl moieties of polyphenols and transition metal ions (e.g., Fe^3+^, Cu^2+^) to form robust supramolecular networks [20,25]. A prevalent fabrication protocol involves interfacial functionalization of metal or metal oxide nanoparticles, in which a polyphenol coating is established via coordination or adsorption to exposed metal sites to enhance dispersion stability. Upon blending with SF, these functionalized nanohybrids initiate in situ supramolecular assembly. Driven by the synergistic interplay of metal–ligand coordination and SF–polyphenol non-covalent interactions (hydrogen bonding/hydrophobic effects), the system evolves into a cross-linked network. This process induces conformational ordering and densification of SF, yielding hierarchical composite architectures devoid of toxic covalent cross-linkers [18]. Notably, the dynamic reversibility of these coordination networks endows the complexes with intrinsic self-healing capabilities, while simultaneously enhancing wet adhesion and antibacterial efficacy [25,120,121].

#### 3.2.3. Photo-Initiated Cross-Linking

Photopolymerization entails the photo-triggered generation of free radicals that drive the covalent cross-linking of functionalized precursors, yielding robust network architectures [122]. By grafting photoreactive moieties onto SF chains, rapid network formation can be achieved under mild conditions with precise spatiotemporal control [123]. This versatility makes the strategy indispensable for advanced fabrication scenarios, including in situ gelation, conformal coating, and high-fidelity 3D printing [124,125]. However, a critical challenge in SF–polyphenol systems is that polyphenols, acting as intrinsic antioxidants, can antagonize polymerization via radical scavenging and competitive light absorption (shielding). This often leads to slowed reaction kinetics, incomplete curing, or structural heterogeneity [126]. To mitigate these effects, the photopolymerization conditions should be rationally optimized according to the optical and redox properties of the incorporated polyphenols. For example, UV-sensitive photo-initiators can be replaced by red-shifted or visible-light-responsive systems, such as riboflavin-mediated photochemistry or the Ru/SPS persulfate system, to reduce spectral overlap with the strong UV absorption of polyphenols and improve light penetration within the hydrogel precursor [127,128]. In addition, increasing the visible-light intensity or extending the irradiation time can enhance radical generation, thereby allowing the crosslinking reaction to better compete with oxygen- and polyphenol-mediated radical quenching [128,129]. For highly polyphenol-loaded formulations, sequential assembly may be particularly useful: the primary SF-based network can first be photo-crosslinked, followed by post-loading or secondary complexation with polyphenols via hydrogen bonding, π–π stacking, or metal–phenolic coordination [130]. This strategy decouples network formation from polyphenol incorporation, thereby reducing radical scavenging and optical shielding during gelation while preserving the reinforcing and bioactive functions of polyphenols in the final network. To reconcile this conflict, it is often judicious to incorporate polyphenols as interfacial functional components rather than high-content bulk phases. Furthermore, optimization strategies such as tailoring the photo initiator system, tuning the light dose, or employing stepwise curing protocols are essential for achieving a refined balance among fabrication efficiency, network homogeneity, and biological safety (Table 5) [18,131].

### 3.3. Comparison and Selection of Fabrication Strategies

There is no single ideal method for combining silk fibroin with polyphenols; the choice depends on application-specific needs and entails trade-offs among interfacial stability, retention of bioactivity, processability, and functional requirements. Physical, non-covalent assembly under mild conditions preserves polyphenol bioactivity (antioxidant/antibacterial), suiting transient uses (e.g., food packaging, short-term dressings) but offers inferior long-term stability compared with covalent crosslinking strategies. Such systems are susceptible to environmental fluctuations, including shifts in pH and ionic strength, as well as prolonged immersion, which can trigger gradual desorption or leaching of active components. Consequently, in scenarios that demand long-term structural integrity or wet-state durability, physical entrapment alone is often insufficient and requires supplementary stabilization protocols [1,7,14].

Compared with non-covalent assembly, covalent crosslinking substantially improves long-term stability and swelling resistance by tethering polyphenols to the SF matrix, thereby enabling durable applications under demanding conditions. However, over-oxidation or extensive crosslinking depletes bioactive phenolic hydroxyl groups, often compromising antioxidant activity [16,47]. Metal–phenolic coordination serves as an effective intermediate strategy that bridges the gap between physical entrapment and covalent fixation. This approach exploits the chelation between metal ions and catechol/galloyl moieties to form bonds with robust binding strength while retaining dynamic reversibility. This unique duality endows the material with pH-stimuli responsiveness, facilitating the development of hydrogels and coatings with controlled release, self-healing, and wet adhesion capabilities [40,42,78]. Nevertheless, incorporating transition metal ions poses challenges related to potential cytotoxicity, ion leakage, and discoloration. Consequently, rigorous safety evaluations are imperative, particularly for food-contact materials or long-term implantable devices [40,134].

Each processing and manufacturing strategy should be carefully considered in light of its intended use. A simple and scalable way to prepare ready-to-use bio-adhesives or injectable hydrogels is to mix the solution using freeze-drying or in situ gelation [18,134,135]. On the other hand, electrospinning is excellent at producing nanofibrous membranes with high specific surface area and biomimetic porosity, which are ideal for gas exchange and cell attachment. However, the complexity and regulatory challenges of organic solvent systems sometimes impede this process [113,114]. Alternatively, impregnation–adsorption is a versatile post-functionalization strategy for preformed scaffolds, though optimizing loading stability often requires secondary treatments (e.g., β-sheet induction) [136]. For applications requiring high spatiotemporal controllability, such as wearable electronics or high-fidelity 3D printing, photo-crosslinking systems (e.g., SFMA) offer highly programmable processing windows [27,123]. In such light-triggered systems, polyphenols are best used strategically as auxiliary interfacial components or post-coatings to enhance wet adhesion and antioxidative capacity, thereby mitigating their potential inhibitory effects on photopolymerization kinetics via radical scavenging [43,115,136].

## 4. Stimuli-Responsive Behaviors of SF–Polyphenol Gels

Generally, the stimulus response of SF–polyphenol systems stems from two types of controllable units. The first involves labile noncovalent interactions, such as hydrogen bonding and electrostatic interactions, which enable network rearrangement and conformational evolution in response to external stimuli. The second comprises dynamic bonding networks, including MPN and redox-active quinone/catechol couples. These units can convert environmental signals (pH, electrical signals, light, etc.) into measurable structural changes, thereby establishing the mechanistic basis for applications in controlled release, reversible adhesion, and bioelectronics [7] (Table 6).

### 4.1. pH Responsiveness

Encapsulation or complexation of polyphenols and other bioactive molecules within SF-based matrices has been explored as an effective strategy to improve their stability and release behavior. Compared with free polyphenols, which are susceptible to oxidation, photodegradation, and rapid loss of activity in physiological or environmental media, SF-based carriers can provide a protective microenvironment and contribute to more sustained release profiles. However, release kinetics should not be attributed solely to SF encapsulation, as they are usually governed by multiple coupled factors, including SF–polyphenol interactions, matrix crystallinity, metal coordination, carrier degradation, and environmental pH. In hydrogel-based delivery systems, pH-responsive swelling, degradation, or network dissociation has been widely used to regulate ion or drug release, although the exact mechanism depends strongly on the specific material composition and network chemistry [137]. For instance, Zhang et al. [138] prepared TA/Zn^2+^-functionalized CaCO_3_ microspheres through an SF-mediated biomineralization strategy for fipronil delivery. This system exhibited pH-selective release behavior, with rapid disassembly and cargo release under acidic conditions while maintaining higher structural stability under alkaline conditions. These findings demonstrate the potential of SF–polyphenol/inorganic hybrid systems for pH-regulated delivery; however, the pesticide-delivery context of this study should be distinguished from biomedical drug delivery, where tissue compatibility, degradation products, pharmacokinetics, and immune responses must also be considered. In biomedical applications, cross-linked SF–proanthocyanidin nanoparticles have been developed as carriers for indocyanine green-mediated photothermal therapy of glioma [68]. Therefore, acid-triggered or microenvironment-responsive release should be regarded as a designable delivery principle rather than a universal mechanism shared by all SF–polyphenol systems. Future studies should further establish quantitative relationships among carrier stability, release kinetics, therapeutic efficacy, and off-target risks under physiologically relevant conditions.

### 4.2. Redox Responsiveness

Polyphenol molecules, enriched with redox-active catechol and galloyl moieties, can confer oxidative microenvironment responsiveness to SF-based complexes. These groups may scavenge ROS through hydrogen atom transfer or single-electron transfer pathways, thereby mitigating oxidative damage during tissue repair [42,47,83]. Simultaneously, their oxidation can generate quinone-like intermediates that react with nucleophilic groups in proteins, potentially contributing to covalent or dynamic covalent reinforcement of SF–polyphenol networks. However, the extent to which such reactions dominate over non-covalent interactions, including hydrogen bonding, hydrophobic association, and physical entanglement, remains insufficiently resolved. EGCG-grafted SF hydrogels, for example, have been reported to be ROS-scavenging wound-dressing materials [47]. This example supports the potential of polyphenol functionalization to integrate antioxidant activity with network modulation, but wound-healing outcomes should be interpreted as system-level responses involving ROS regulation, modulation of inflammation, matrix architecture, degradation behavior, and cell–material interactions rather than as direct proof of a single oxidation-triggered cross-linking mechanism.

By constructing metal–polyphenol coordination architectures, the system’s antioxidant behavior is elevated from passive radical scavenging to active, enzyme-mimetic regulation. For instance, Cao et al. [83] engineered copper–TA (CuTA) nanozymes within an SF hydrogel matrix (CuTA@SF). It retains a significant direct clearance capacity (approximately 60% DPPH inhibition), and the true efficacy of this system lies in its cellular regulatory effect. Under simulated inflammatory (IL-1β) or oxidative (H_2_O_2_) stress, the composite effectively reduced intracellular ROS levels by activating the Nrf2 signaling pathway and upregulating downstream antioxidant genes (e.g., SOD-1, HO-1), thereby reinforcing endogenous defense mechanisms. This bioactive regulation acts synergistically with the material’s mechanics: the abundant reversible SF–polyphenol complexes provide dynamic energy dissipation, ensuring the wet-state structural stability required for demanding tissue repair applications, such as bone and cartilage regeneration [42,139].

### 4.3. Electrical Responsiveness

Introducing electrical responsiveness is a key strategy for endowing SF–polyphenol complexes with intelligence. This approach allows for precise interaction with external fields or endogenous bioelectricity by combining the redox activity of polyphenols with the intrinsic piezoelectric response and the primary ion-migration conductivity of hydrated SF. It is important to clarify that hydrated SF conducts charge predominantly via ion migration, such as H^+^, Na^+^, and Cl^−^ migration, rather than through delocalized electronic conduction. A study indicates that hydrated SF exhibits intrinsic ion-migration conductivity and piezoelectricity under mechanical load [27]. Upon integration, polyphenols regulate SF chain packing via non-covalent interactions, such as π–π stacking and hydrogen bonding, thereby creating optimized hydrated ion-transport pathways that enhance the system’s ionic conductivity [30,140]. More importantly, polyphenol moieties, such as catechol and galloyl groups, act as redox switches. Specifically, the reversible conversion between catechol/galloyl and quinone states involves coupled electron and proton transfer, thereby introducing localized redox-mediated charge-transfer sites within the ion-conducting SF matrix. This redox activity not only enables charge transfer under external electrical stimulation but also modulates the local proton concentration and ionic environment, thereby coupling ion migration with localized redox-mediated electron transfer. The apparent charge-transport behavior may involve coupled ionic and redox-mediated electronic contributions, rather than pure ion migration alone. This redox-mediated charge-transport mechanism is crucial for maintaining the redox balance and functional stability of the material during extended operation [141].

### 4.4. Thermal Responsiveness

Temperature is a critical external trigger that modulates the supramolecular assembly of SF–polyphenol complexes. Thermodynamically, elevated temperatures intensify the hydrophobic association of SF chains, promoting the conformational transition towards β-sheet-rich structures and accelerating network gelation [142]. The incorporation of TA introduces extensive hydrogen bonding and additional hydrophobic interactions. These non-covalent forces are inherently temperature-labile, thereby synergistically regulating the gelation kinetics and the ultimate stability of the network [55]. Leveraging this property, hydrogels with rapid sol–gel transitions at physiological temperatures have been engineered, rendering them ideal for injectable, in situ scaffolds and bioadhesives [25,143]. Furthermore, the thermally induced structural relaxation and energy dissipation within the network provide a mechanism for on-demand cargo delivery. Consequently, thermal responsiveness is frequently used to trigger drug release and is often integrated with photothermal therapy for precision medicine [143,144].

### 4.5. Light Responsiveness

**Figure 3:**
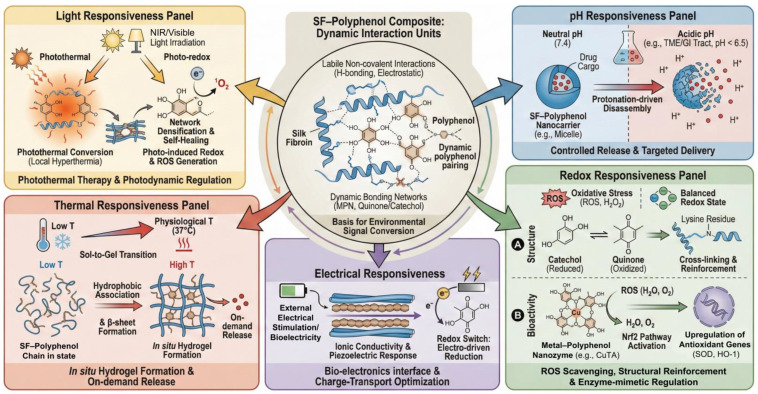
Stimuli-responsive strategies for intelligence-imparted SF–polyphenol complexes.

Light acts as an external trigger for SF–polyphenol composites via photothermal conversion and photo-induced redox reactions. Catechol/galloyl moieties and their metal complexes serve as chromophores that, under NIR/visible irradiation, convert absorbed photons primarily into localized heat through non-radiative decay [20,54,145]. In such photothermal systems, NIR-induced local hyperthermia promotes the thermal relaxation of SF chain segments by increasing chain mobility and partially loosening transient hydrogen bonds and surrounding hydration shells. This relaxation process enables kinetically trapped or hydrated SF conformations to overcome local energy barriers and rearrange into more thermodynamically favorable β-sheet crystalline domains. The resulting β-sheet-rich crystalline regions serve as physical crosslinking points, thereby enhancing conformational ordering and network densification. Concurrently, the reversible non-covalent interactions (hydrogen bonding/hydrophobic effects) afforded by polyphenols provide dynamic structural plasticity, enabling localized self-healing or remodeling while maintaining macroscopic integrity [20,54]. Beyond thermal effects, photo-irradiation can induce charge separation in photosensitive polyphenol motifs, generating ROS for photodynamic sterilization or therapy. Crucially, the intrinsic redox reversibility of polyphenols establishes a dynamic buffering mechanism that balances ROS activation and the prevention of oxidative stress [145]. Furthermore, in photo-initiated cross-linking, light triggers rapid SF gelation, in which polyphenols not only stabilize the nascent network but also impart antioxidant and anti-inflammatory bioactivities. This spatiotemporal controllability positions light-responsive SF–polyphenol complexes as powerful platforms for in situ tissue engineering and precision therapeutics (Figure 3) [90].

## 5. Applications of SF–Polyphenol Gels and Hydrogel-Derived Materials

**Figure 4:**
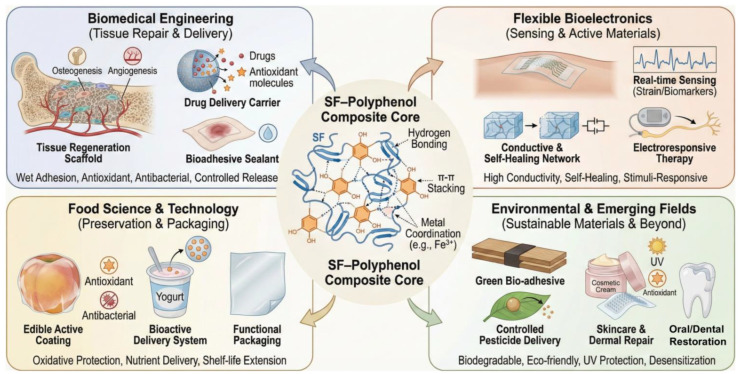
Representative applications of polyphenol-functionalized natural polymer materials, as illustrated by SF–polyphenol systems. Related functional outcomes are also widely observed in polysaccharide-based material platforms.

Recently, the complex of SF and polyphenols has emerged as a pivotal research direction in functional biomaterials. Driven by a rich repertoire of molecular interactions (spanning hydrogen bonding, π–π stacking, and metal ligand coordination), these complexes exhibit exceptional interfacial wet adhesion, alongside intrinsic antioxidant, antibacterial, and stimuli-responsive functionalities. Capitalizing on these molecular-level synergies, research has proliferated across the biomedical spectrum, ranging from tissue regeneration and therapeutic delivery to bio-adhesives. Moreover, the versatility of SF–polyphenol complexes has expanded their utility into flexible bioelectronics (e.g., epidermal sensing) and industrial sectors such as food preservation and environmental remediation. The following sections provide a comprehensive overview of the latest breakthroughs in these diverse application landscapes (Figure 4). Notably, many of these functional outcomes, particularly film formation, barrier enhancement, controlled release, and active packaging, are also shared by polysaccharide–polyphenol systems, reinforcing the broader relevance of polyphenol-mediated assembly in natural polymer materials.

### 5.1. Bioadhesive and Biointerface-Related Gels

Biomedical and biointerface-related applications of SF–polyphenol systems can be broadly understood through three recurring design logics: (i) wet-adhesion-dominant systems, in which catechol/galloyl-rich polyphenols may enhance interfacial bonding between the material and hydrated tissue interfaces under specific conditions; (ii) redox- and bioactivity-regulated systems, in which polyphenols participate in network stabilization or structural regulation while further introducing antioxidant, antibacterial, or anti-inflammatory functions; and (iii) delivery-oriented systems, in which polyphenol-mediated interactions regulate loading, retention, and release behavior. This classification helps connect molecular interaction modes with application-specific functional outputs, rather than viewing individual studies as isolated demonstrations. It should be noted that the dominant mechanisms vary across systems, and their functional performance often depends on the type of polyphenol, SF conformational changes, crosslinking strategy, material format, and the specific biological environment. As this section focuses on bioadhesives and biointerface-related gels, the following discussion primarily addresses wet adhesion, regulation of the wound/soft-tissue microenvironment, and integration with bone or osteochondral interfaces; delivery-oriented systems are discussed separately in the subsequent section.

Within this framework, SF serves as a processable and structurally adaptable matrix, whereas polyphenols act as multifunctional regulators through hydrogen bonding, hydrophobic interactions, π–π stacking, and metal–phenolic coordination. These interactions improve interfacial adhesion, matrix cohesion, antioxidant stability, and responsiveness, thereby enabling the development of scaffolds, sealants, delivery platforms, and soft active materials. Importantly, the relative importance of each interaction mode depends on the target function: wet adhesion generally relies on interfacial catechol chemistry and dynamic bonding, whereas delivery systems place greater emphasis on encapsulation stability, environmental responsiveness, and controllable network disassembly.

In systems related to wet adhesion and tissue–interface fixation, the function of polyphenols is generally associated with their abundant phenolic hydroxyl groups, dynamic noncovalent interactions, and potential metal–phenol coordination. Inspired by mussel-like interfacial chemistry and tannic-acid-mediated supramolecular assembly, the incorporation of small polyphenols into SF matrices has enabled multifunctional material systems with enhanced wet adhesion, antioxidant stability, antibacterial activity, and controllable degradation [24,43,47,66,113]. Previous studies have shown that TA/SF-based medical adhesives can exhibit adhesion and mechanical advantages in wet tissue environments. Bai et al. [139] further extended this strategy to hard tissue interfaces, employing TA as a phenolic adhesive molecule to co-assemble with SF and hydroxyapatite into an SF@TA@HA mineral–organic hybrid hydrogel. This system is designed for fracture fixation and early bone regeneration in the moist bone tissue environment. Its core advantage lies not merely in adhesion but also in the organic support provided by SF, the mineral phase contributed by HA, and the interfacial bonding and energy dissipation mediated by TA. It should be noted that bone adhesives, soft-tissue sealants, and skin-adhesive dressings encounter markedly different interfacial compositions, hydration states, and mechanical environments; therefore, adhesion mechanisms identified in one system should not be directly generalized to all biological interfaces.

In wound and soft-tissue repair, SF–polyphenol systems primarily function by integrating structural support with local microenvironmental regulation. Polyphenol functionalization not only improves the network stability of SF-based materials but also introduces functions such as ROS scavenging, antibacterial activity, anti-inflammatory effects, and immunomodulation in specific systems [47,66]. In addition, Ag/GA-loaded methacryloyl SF hydrogels have been reported to promote diabetic wound healing through combined antibacterial activity, ROS scavenging, regulation of macrophage polarization, and pro-angiogenic effects [50]. Based on the defects of pure sodium alginate, such as unstable mechanical properties and lack of bioactivity, Reza Eivazzadeh-Keihan et al. [146] introduced TA, SF, and Fe_3_O_4_ magnetic nanoparticles into the sodium alginate matrix. Through calcium-ion crosslinking, they significantly improved the mechanical properties and biocompatibility of the sodium alginate hydrogel, thereby developing a multifunctional nanocomposite hydrogel (SA-TA/SF-Fe_3_O_4_) suitable for wound dressings. Compared with pure sodium alginate hydrogels, the mechanical properties of the SA-TA/SF-Fe_3_O_4_ hydrogel were significantly enhanced by SF crosslinking. FE-SEM revealed a uniform porous structure with a pore size of 80–90 nm. At a concentration of 2 mg/mL, the red blood cell viability was nearly 100% (in compliance with the ISO 10993-5 standard [147]), and the viability of normal cells (HEK293T) exceeded 93% after 72 h. Meanwhile, the introduction of TA endowed the material with antioxidant function. Hemolysis and MTT assays further confirmed its applicability as a wound dressing. These studies suggest that the role of SF–polyphenol systems in wound repair is better understood as a synergistic outcome of material architecture, anti-infective capacity, oxidative stress regulation, and host–cell responses, rather than being simply attributed to a specific crosslinking mechanism or a single antioxidant effect.

In bone and cartilage regeneration, research on SF–polyphenol systems has generally shifted from soft-tissue adhesion toward interfacial integration, mineralization regulation, anti-infective protection, and modulation of the tissue-regenerative microenvironment. In these applications, polyphenols can serve not only as components that mediate interfacial interactions or regulate network structures but also as active agents that remodel the local microenvironment through metal–phenol coordination, nanozyme activity, or redox regulation. EGCG-loaded electrospun SF/PCL nanofibrous membranes have been used to enhance guided bone regeneration, suggesting that EGCG can function as an active component in the design of bone-repair membranes [113]. TA-induced mineral–organic bone adhesives use TA as a phenolic adhesive component that co-assembles with SF and hydroxyapatite to form inorganic–organic hybrid hydrogels, showing potential for fracture fixation and early bone regeneration in moist biological environments [139]. These studies indicate that polyphenol integration enhances SF scaffolds not only by improving structural stability, wet adhesion, and interfacial integration but also by modulating oxidative stress, infection, inflammation, and tissue-regenerative microenvironments. Therefore, the resulting repair effects should be viewed as system-level outcomes driven by material architecture, mechanical support, interfacial interactions, bioactive component release, and host cellular responses, rather than by a single crosslinking, antioxidant, and mineralization regulation.

### 5.2. Delivery-Oriented Gels and Hydrogel Platforms

To circumvent the intrinsic limitations of natural SF for therapeutic delivery, researchers have leveraged the abundant phenolic hydroxyl groups in polyphenols to engineer multifunctional platforms through hydrogen bonding, π–π stacking, and metal coordination. Early efforts focused on enhancing payload stability and release kinetics. Qi et al. [32] employed tyrosinase-catalyzed coupling of catechins to SF surfaces, thereby significantly boosting antioxidant stability and providing a green synthetic route for protein carriers. In practical applications, the most compelling direction for SF–polyphenol hydrogels is not to address all potential diseases but to enable localized delivery in moist, dynamic, or infection-prone microenvironments. Taking oral mucosal delivery as an example, Cheng et al. [148] designed a bilayer SF-TA adhesive microneedle patch that combines an SF microneedle layer with an SF–TA wet-adhesive layer for the transmucosal delivery of triamcinolone acetonide. In this system, the mechanical strength of a single needle reached 0.071 newtons, enabling penetration of the oral mucosa; the drug was continuously released for at least 7 days, and the wet adhesion strength reached 37.74 kPa, approximately sevenfold higher than that of a commercial oral patch. Similarly, the ternary SF/wool keratin/TA hydrogel developed by Jafari et al. exhibited in situ gelation, self-healing, 3D printability, antibacterial activity, and antioxidant properties, with G′ exceeding 100 kPa, and promoted wound healing in a full-thickness skin defect model [67]. However, the tissue-repair effects observed in this study may arise from the combined contributions of polyphenol-mediated antioxidant activity, keratin-derived cell-adhesion sites, and the mechanical support provided by the gel; therefore, the therapeutic outcome should not be attributed solely to a single polyphenol adhesion mechanism.

Infected wounds are among the most relevant application scenarios for SF–polyphenol hydrogels as delivery-oriented hydrogel platforms. Zeng et al. [149] constructed a GS@EG-Cu-CA NPs hydrogel composed of thiolated gelatin, methacrylated SF, and EGCG–Cu–κ-carrageenan metal–polyphenol nanoparticles for the treatment of MRSA-infected wounds; this system was designed as a localized therapeutic platform integrating antibacterial activity, antioxidant capacity, and catalytic NO release. Su et al. [145] developed a Curcumin/ZnMOF/SF@Gel (Cur) smart dressing by embedding curcumin-loaded zinc-doped ferricyanide-derived MOF into a gelatin/SF crosslinked hydrogel; upon white-light activation, this system generates ROS for rapid bacterial killing while scavenging excess ROS through the enzyme-like antioxidant activity of ZnMOF and the polyphenolic structure of curcumin. Cur release from this system was quantitatively evaluated under conditions including room temperature in the dark, 37 °C in the dark, and white-light irradiation, with three experimental replicates (n = 3); additionally, the hydrogel state was examined over the range of 25–50 °C, demonstrating temperature- and light-responsive delivery characteristics. Therefore, the advantage of metal–polyphenol–SF hydrogels lie not in simple drug encapsulation but in integrating antibacterial action, NO/ROS regulation, and infected-microenvironment intervention into localized delivery strategies through wet-interface retention, dynamic coordination networks, and stimulus-responsive processes.

### 5.3. Flexible Bioelectronics and Conductive Gels

Achieving long-term, stable, and high-fidelity monitoring of flexible physiological signals is an important goal of skin-integrated wearable electronics. However, conventional hydrogel sensors often struggle to simultaneously meet the requirements of high stretchability, wet adhesion, conductive stability, self-healing capability, and biocompatibility. While SF provides exceptional mechanical flexibility, dielectric stability, and biodegradability, polyphenolic molecules (abundant in catechol moieties) establish dynamic conductive networks with SF segments through hydrogen bonding, π–π stacking, and metal phenolic coordination. This intricate web of supramolecular interactions not only bolsters the wet adhesion and mechanical coupling of SF but also imparts electronic conductivity and stimuli responsiveness. Consequently, these complexes are ideal candidates for constructing skin-adhesive sensors, self-powered systems, and implantable platforms for controlled therapeutic release [30,91,150].

In conductive hydrogel sensors, the key role of the SF–polyphenol network is to maintain continuous, stable, and dynamically recoverable electronic or ionic transport pathways. For sensing applications in underwater or highly humid environments, oxidative deactivation of conductive fillers and interfacial debonding are major factors limiting the long-term stability of devices. Wang et al. [91] developed a TA-crosslinked MXene/SF conductive hydrogel for diving training and underwater health monitoring, in which TA stabilized MXene nanosheets through antioxidant effects, coordination via phenolic hydroxyl groups, and hydrogen bonding with SF, thereby mitigating MXene oxidative deactivation in aqueous and oxygen-containing environments and maintaining underwater strain-sensing performance. This system was applied to diving posture correction, underwater health monitoring, and safety warning, indicating that the SF–TA network can provide wet-state stabilization for MXene conductive pathways. In conductive polymer systems, Zheng et al. [121] introduced polypyrrole (PPy) into an SF/TA dynamic network to fabricate an SF/TA/PPy conductive hydrogel with stretchability, wet adhesion, self-healing capability, and biocompatibility. This hydrogel enabled the underwater recovery of mechanical, electrical, and sensing performance and was used to monitor large-amplitude motions of the fingers, wrists, elbows, and knees, as well as small-strain physiological signals such as smiling, frowning, coughing, and breathing; moreover, its signal responses could be used for underwater Morse-code communication. In ionically conductive systems, PVA/borax/SF/TA (PBST) hydrogels form multiple dynamic crosslinked structures via PVA–borax dynamic borate ester bonds and SF/TA hydrogen-bonding networks, exhibiting self-adhesion, self-healing, stretchability, and biocompatibility for monitoring human motion and subtle physiological signals [140]. Compared with composites that rely solely on percolated networks of conductive fillers, these systems share a common feature: polyphenol-mediated dynamic interactions can reconstruct after stretching, compression, or damage, thereby restoring the hydrogel network and conductive pathways and improving device stability in humid environments and under repeated deformation.

Beyond strain sensing, SF–polyphenol composite systems can be further extended to skin-conformal soft electrodes and electrostimulation-based therapeutic platforms. PVA/SF/TA/GR hydrogels achieve high stretchability, rapid response, and post-damage functional recovery through the synergistic construction of SF–TA dynamic hydrogen-bonding networks and graphene conductive pathways. Zhao et al. [151] reported a high-performance PVA/SF/TA/GR sensor with a stretchability exceeding 6000%, a response/recovery time of 12.5 ms, and a strain-detection range greater than 400%, demonstrating its potential for wearable electronics and human–machine interaction applications. The combination of SF and TA improves the self-healing ability, mechanical performance, and viscoelasticity of the hydrogel, while graphene serves as the conductive component to establish electrical-signal transmission networks, thereby maintaining stable sensing responses under large deformation. For skin-conformal bioelectrodes and electroactive therapeutic platforms, Zhang et al. [152] demonstrated a polydopamine (PDA) nanoclay SF electrode that can detect subtle signals such as pulse and serve as an epidermal soft electrode for recording electrophysiological signals, including electrocardiographic and electromyographic signals. In this system, the catechol groups of PDA enhance wet interfacial adhesion, whereas the SF-based hydrogel network provides flexible support that matches the mechanical properties of skin, thereby reducing the contact instability typically associated with conventional rigid electrodes during dynamic motion. Ran et al. [141] introduced polydopamine–iron/poly(3,4-ethylenedioxythiophene)/silk fibroin (PDA-Fe-PEDOT) conductive nanozymes into a fish gelatin/methacrylated SF hydrogel to obtain a composite hydrogel integrating conductivity, redox-regulating capability, and electrostimulation responsiveness, which was combined with vagus nerve electrical stimulation for diabetic wound repair. Compared with conventional flexible sensors, the functional focus of these systems has shifted from passive signal acquisition to active electrical regulation and intervention in the pathological microenvironment. Their material-design rationale is to couple electronic conduction, antioxidant regulation, and electrostimulation responsiveness through PDA-Fe-PEDOT conductive nanozymes, thereby promoting inflammatory modulation and tissue regeneration in chronic wounds.

### 5.4. Food-Related, Sustainable, and Environmentally Responsive Gel Systems

In the field of food science, the development of advanced materials capable of achieving natural oxidative protection, efficient bioactive delivery, and functional active packaging is a critical step toward improving food quality and ensuring nutritional health. The performance of traditional food ingredients and packaging materials has gradually become inadequate to meet the growing demands for stability, bioavailability, and preservation. SF possesses exceptional biocompatibility and film-forming capabilities, enabling the assembly of stable composite architectures with polyphenols via hydrogen bonding and hydrophobic interactions. This integration significantly bolsters the antioxidant potency and environmental resilience of the phenolic compounds. Hcini et al. [153] encapsulated Tunisian rosemary extracts within SFNs, maintaining a radical-scavenging rate exceeding 85% at 37 °C, underscoring the efficacy of this platform for oxidative defense. Addressing nutrient bioavailability, Paladines Quezada et al. [154] demonstrated that red wine polyphenols loaded into SFNs exhibit robust structural integrity and controlled-release profiles during simulated gastrointestinal digestion, positioning these complexes as superior delivery vehicles for functional food ingredients. Furthermore, SF–polyphenol complexes in the form of membranes or microspheres offer substantial value for active packaging. Ma et al. [155] reported that films engineered from SF–TA complexes exhibited enhanced mechanical durability and potent antibacterial activity, making them ideal for edible coatings or preservative barriers. Food-functional materials based on SF–polyphenol composite systems can synergistically optimize antioxidant protection, bioactive compound delivery, and green packaging, providing an important material basis for the development of safe, biodegradable, and bioactive materials in food science.

In the field of environmentally friendly and sustainable materials, developing green functional materials that combine high performance, low toxicity, and degradability is an important strategy to reduce reliance on conventional petrochemical-based materials and promote sustainable development. Conventional synthetic resins, adhesives, and functional coatings often exhibit formaldehyde emissions, poor degradability, and high environmental burdens, making them increasingly unable to meet the requirements of green manufacturing and ecological safety. The synergy between SF and polyphenols can not only enhance mechanical strength, wet adhesion, antioxidant performance, and interfacial bonding but also preserve the bio-based, nontoxic, and environmentally friendly characteristics of the materials, thereby providing an important material basis for the development of renewable adhesives, functional membranes, and protective coatings. Ma et al. [156] constructed a PDA functional layer on the SF surface via in situ dopamine polymerization, yielding an SF/PDA functional component that was subsequently incorporated into a soybean-based biomass adhesive system. This system achieved excellent cold-pressing adhesion performance through multiple hydrogen-bonding crosslinks among PDA phenolic hydroxyl groups, SF protein segments, and matrix proteins, together with the exclusion of interfacial water layers. The cold-pressing bonding strength reached 771.0 kPa, representing a 267.1% increase compared with the unmodified soybean-based adhesive; the toughness reached 1.54 MJ m^−3^, corresponding to a 208.0% increase. In addition, the synergistic interaction between borate and phenolic hydroxyl groups further endowed the adhesive with mildew resistance and flame-hindering properties, as evidenced by the absence of mildew after 20 days of storage and combustion-inhibiting behavior in burning tests. Therefore, polyphenol- or catechol-functionalized SF can not only enhance the interfacial adhesion and cohesive strength of biomass adhesives but also improve their durability and ecological safety, providing an effective design strategy for degradable wood adhesives and green packaging materials.

Beyond the primary sectors discussed, SF–polyphenol complexes exhibit extensive versatility across several emerging domains. In skincare and dermal repair, integrating SF with polyphenols such as EGCG and TA provides superior antioxidant, anti-inflammatory, and ultraviolet protection. These systems have been successfully used to engineer absorbable masks, microneedle arrays, and postoperative recovery gels that provide skin-whitening, soothing, and desensitizing effects while maintaining excellent biocompatibility and sustained active release [40,55,73]. For oral and dental restorations, SF–polyphenol complexes significantly enhance the stability of sealants and antimicrobial dressings on gingival and dentin surfaces by improving film-forming efficiency and wet tissue adhesion. Research indicates that these materials facilitate deep occlusion of the dentinal tubules and inhibit oral cariogenic bacteria, thereby mitigating dentin hypersensitivity and accelerating oral epithelial regeneration [147,157,158,159]. In the realm of sustainable agriculture, ternary microspheres composed of SF, polyphenols, and metal ions have been developed as stimuli-responsive pesticide carriers. These platforms achieve the controlled release of active ingredients by modulating pH or the metal–phenolic coordination state. This strategy has been effectively applied to deliver pesticides such as fipronil, thereby demonstrating superior foliar adhesion, rapid biodegradability, and enhanced environmental safety [138]. Collectively, these cross-disciplinary applications underscore the immense potential of the SF–polyphenol complexes as a multifunctional, biocompatible material platform for diverse industrial and clinical applications.

## 6. Challenges and Future Perspectives

The central limitation of the current literature is that many studies optimize a single desirable output, such as adhesion, ROS scavenging, or injectability, without reporting the countervailing penalties in gelation kinetics, network homogeneity, retained phenolic content, degradation, or long-term wet stability. Future studies should therefore report standardized figures of merit under comparable conditions, including SF concentration and molecular weight, polyphenol feed and retained content, pH, ionic strength, gelation time, G′/G″, adhesion substrate, swelling, release/leaching, antioxidant retention, and cytocompatibility.

Despite the rapid progress in polyphenol-functionalized natural polymer materials, several fundamental and translational challenges still limit their broader development and practical implementation. Although significant advances have been made in understanding polyphenol-mediated assembly in SF-based systems, many of the key issues identified here are equally relevant to polysaccharide-based matrices, including cellulose, chitosan, alginate, pectin, and starch. Addressing these challenges is essential not only to improve the reproducibility and performance of current systems but also to establish general design principles for next-generation carbohydrate-relevant functional materials.

### 6.1. Polyphenol Stability and Oxidation Control

One of the most persistent challenges in polyphenol-functionalized materials is the intrinsic chemical instability of polyphenols. Many polyphenols are highly sensitive to oxygen, light, pH, temperature, and metal ions, and can therefore undergo oxidation, auto-polymerization, structural rearrangement, or degradation during processing and storage. This instability creates a fundamental contradiction in material design. On the one hand, polyphenols are introduced to provide antioxidant, adhesive, or crosslinking functions; on the other hand, their uncontrolled oxidation may reduce the availability of phenolic hydroxyl groups, alter intermolecular interaction profiles, and cause batch-to-batch variation in final material properties.

In SF–based systems, oxidation can strongly affect supramolecular assembly, conformational transitions, and network stability. For example, catechol- or gallol-containing polyphenols may initially promote hydrogen bonding and non-covalent association, but excessive oxidation can shift the system toward irreversible covalent crosslinking or heterogeneous aggregation. Similar phenomena are also expected in polysaccharide-based systems, particularly in chitosan- and pectin-containing matrices, where quinone formation may lead to uncontrolled secondary reactions with amino or hydroxyl groups. As a result, the same polyphenol may exert beneficial or detrimental effects depending on processing history, environmental exposure, and matrix composition.

Future research should therefore pay greater attention to oxidation control during material preparation and storage. Rational strategies may include deoxygenated processing, pH regulation, antioxidant co-stabilizers, microenvironment engineering, encapsulation-assisted protection, and controlled metal coordination. More importantly, polyphenol stability should not be discussed only in qualitative terms. Quantitative characterization of oxidation state, residual phenolic activity, and time-dependent structural evolution should become a routine component of material evaluation. Such efforts would help clarify how much of the observed structure–property relationship arises from intact polyphenols, oxidation intermediates, or secondary polymerization products.

### 6.2. Reproducibility and Processing Scalability

A second major limitation lies in the reproducibility and scalability of current preparation methods. Many reported polyphenol-functionalized materials are fabricated under laboratory-scale conditions using highly specific concentrations, mixing sequences, pH windows, solvent conditions, and curing protocols. However, because polyphenol-mediated interactions are highly dynamic and multivariate, even subtle differences in preparation procedures can lead to pronounced changes in assembly kinetics, phase behavior, crosslinking density, and material morphology. This sensitivity is especially evident in SF-based systems, where polyphenols can regulate the random coil-to-β-sheet transition and thereby alter gelation, aggregation, and long-term stability. Similar processing sensitivity is also common in polysaccharide systems, where hydration state, ionic strength, and chain conformation strongly influence polyphenol binding.

Another source of poor reproducibility comes from the diversity of polyphenol feedstocks. Even when the same nominal polyphenol is used, differences in purity, source, degree of polymerization, isomer composition, and residual impurities may affect interaction behavior and material performance. This issue is particularly important for plant extracts or partially purified polyphenol fractions, which are attractive from a sustainability perspective but often chemically heterogeneous. Without adequate compositional characterization, it becomes difficult to establish transferable structure–property relationships across studies.

From an engineering standpoint, the transition from proof-of-concept formulations to scalable manufacturing remains underdeveloped. Although electrospinning, photo-crosslinking, impregnation, and in situ gelation have shown versatility at the laboratory scale, their industrial translation requires more rigorous process-level control over viscosity, gelation/curing kinetics, solvent compatibility, component stability, and product uniformity. Therefore, in-line or in-process characterization tools, particularly viscometry and rheological monitoring, should be implemented as standard quality-control strategies for industrial-scale protein–polyphenol assembly. By continuously tracking gel-point formation, G′/G″ evolution, curing completion, and premature aggregation, these tools would enable timely adjustment of shear rate, temperature, light dose, pH, or component concentration, thereby improving batch-to-batch consistency and reproducibility during scale-up. Therefore, future studies should move beyond descriptive demonstrations and provide more standardized processing windows, critical formulation parameters, and scalability-relevant metrics. Establishing reproducible protocols and reporting standards will be essential to enable meaningful comparisons across different natural polymer platforms.

### 6.3. Performance Trade-Offs in Multifunctional Gel Design

Although this review uses SF-based assemblies as a representative model, one of the most important future directions is to bridge insights from protein- and polysaccharide-based systems. At present, these two material classes are often discussed separately, even though many of the same interaction principles govern polyphenol-mediated assembly in both. Hydrogen bonding, hydrophobic association, metal–phenolic coordination, and oxidation-triggered covalent reactions are not unique to proteins; they also operate extensively in carbohydrate-based matrices. However, the manifestation of these interactions depends strongly on the chemical architecture of the host polymer, including chain rigidity, functional-group density, crystallinity, hydration behavior, and charge distribution.

For example, in SF systems, polyphenols may trigger conformational switching, induce β-sheet formation, and reinforce hierarchical networks through multipoint binding. In contrast, in polysaccharide matrices, polyphenols more often regulate interchain cohesion, water sensitivity, film compactness, and interfacial barrier behavior. These differences should not be viewed as barriers but as opportunities to establish broader comparative frameworks. Instead of treating protein and polysaccharide systems as isolated cases, future research should identify common descriptors that explain polyphenol-mediated behavior across different natural polymers. Such descriptors may include polyphenol hydroxyl density, aromaticity, redox activity, molecular weight, binding-site accessibility, polymer chain mobility, and local hydration microenvironment.

Bridging these design principles would be especially valuable for hybrid systems that combine proteins and polysaccharides in a single material platform. These multicomponent matrices can potentially integrate the conformational adaptability of proteins with the film-forming, barrier, or rheological advantages of polysaccharides. Polyphenols may then act as interfacial mediators that couple these components into more integrated supramolecular architectures. Developing such cross-platform design logic is likely to become an important direction for advanced hydrogels, coatings, adhesives, films, and delivery systems.

### 6.4. Toward Predictive Design of SF–Polyphenol Gels

Beyond individual formulations, the field now needs more generalized and predictive frameworks for designing polyphenol-functionalized natural polymer materials. At present, many studies remain empirically driven, focusing on whether incorporating a selected polyphenol improves a given property in a specific system. While such studies are valuable, they often do not reveal why one polyphenol performs better than another, or how processing, molecular structure, and matrix composition interact to determine final function. As a result, progress remains fragmented, and knowledge transfer across different material systems remains limited.

A key future goal should therefore be the development of integrated structure–processing–property frameworks. In such a framework, polyphenol molecular features would be correlated with interaction modes, network evolution, and final material performance across both protein- and polysaccharide-based matrices. This would facilitate more rational selection of polyphenols for targeted applications, such as improving mechanical reinforcement, wet adhesion, antioxidant retention, controlled release, barrier performance, or stimuli responsiveness. In the context of carbohydrate polymers, such frameworks could help identify which classes of polyphenols are best suited for cellulose-based films, chitosan-based hydrogels, alginate-based carriers, or starch-based active packaging systems.

To achieve this goal, future studies should increasingly combine molecular spectroscopy, thermodynamic analysis, rheology, advanced imaging, and computational modeling. Data-driven strategies and machine-learning-assisted materials design may also become valuable tools for mapping multidimensional relationships among composition, processing conditions, and performance outputs. Equally important is the need for standardized comparative studies using well-defined polyphenol families across multiple natural polymer matrices. Such work would generate the systematic datasets needed to move the field from case-by-case optimization toward predictive materials engineering.

Overall, the next stage of development should aim not merely to produce more polyphenol-modified materials but to establish transferable design rules for natural polymer systems, particularly for carbohydrate polymers. By integrating mechanistic understanding, processing control, and cross-platform comparison, future research can advance polyphenol-functionalized natural polymers from promising laboratory concepts to robust, scalable, and application-oriented material technologies.

### 6.5. Industrial Translation and Regulatory Landscape

Beyond predictive material design, the next major challenge for polyphenol-functionalized natural polymers is to establish translation-oriented frameworks that connect laboratory formulations with scalable, reproducible, and regulatory-compliant products. At present, many systems remain at the proof-of-concept stage, where performance is demonstrated under carefully controlled laboratory conditions but rarely evaluated under manufacturing, sterilization, storage, and regulatory constraints. This gap is particularly important for SF and polysaccharide-based biomaterials, because their final properties are governed not only by molecular composition but also by processing history, hydration state, conformational transitions, and post-processing treatments. For silk-based medical devices, previous translational studies have emphasized that successful clinical translation requires the integrated evaluation of manufacturing robustness, quality requirements, biological safety, toxicological risk, and functional performance rather than material performance alone.

Therefore, future studies should move from formulation-level optimization toward process–quality integration. For polyphenol-functionalized natural polymers, critical process parameters may include polymer molecular weight distribution, polyphenol purity and oxidation state, solution viscosity, pH, ionic strength, oxygen exposure, light penetration, temperature, drying conditions, and sterilization method. These variables can directly affect gelation or curing kinetics, hydrogen-bonding density, metal–phenolic coordination, π–π stacking, β-sheet formation in protein matrices, and network homogeneity, ultimately determining gelation time, storage modulus, swelling behavior, degradation rate, adhesive strength, release kinetics, and bioactivity retention. Accordingly, standardized quality-control criteria should be established for different product categories, including viscosity evolution, injectability, gel-point formation, G′/G″ development, curing completion, adhesive strength, thickness uniformity, barrier performance, antioxidant retention, migration behavior, endotoxin burden, extractables/leachables, and post-sterilization performance retention. Sterilization is particularly important because silk fibroin biomaterials are sensitive to post-processing conditions; autoclaving has been reported to increase scaffold stiffness and reduce degradation rate, whereas γ-irradiation can accelerate scaffold degradation, and ethylene oxide may affect cell response if residues are not adequately removed. Thus, sterilization should be treated as a formulation-dependent design parameter rather than a universal downstream procedure.

From a regulatory perspective, the classification of polyphenol-functionalized natural polymer products will depend strongly on intended use, mechanism of action, contact duration, and risk profile. Simple wound dressings, hemostatic materials, tissue adhesives, or resorbable scaffolds may be regulated primarily as medical devices, whereas systems delivering drugs, growth factors, living cells, or other biologically active agents may enter combination-product or regenerative-medicine pathways. The Food and Drug Administration (FDA) defines combination products as products composed of two or more regulated components, including drug/device, biologic/device, drug/biologic, or drug/device/biologic combinations. For medical devices, ISO 10993-based biological evaluation provides a useful framework because it emphasizes risk-based assessment according to material composition, tissue contact, and exposure duration [160]. A relevant precedent is the silk fibroin-based SERI Surgical Scaffold, which was FDA-cleared as a sterile, single-use, long-term bioresorbable scaffold for soft-tissue support and repair. Overall, future research should not only generate new functional formulations but also build transferable translation rules that integrate molecular design, process control, quality attributes, sterilization compatibility, biological safety, and regulatory classification, thereby moving polyphenol-functionalized natural polymers from promising laboratory concepts toward robust, scalable, and application-ready material technologies.

## 7. Conclusions

In summary, SF–polyphenol systems represent a versatile class of gels and hydrogels in which supramolecular interactions and dynamic crosslinking govern gelation, structural evolution, and multifunctional performance. This review has highlighted how hydrogen bonding, hydrophobic interactions, π–π stacking, metal coordination, and covalent crosslinking regulate network formation, responsiveness, and structure–property relationships in SF–polyphenol gel systems. By integrating mechanistic understanding with gelation strategies, characterization methods, and application-oriented design, SF–polyphenol assemblies provide a useful framework for developing multifunctional gels with tunable mechanics, wet adhesion, antioxidant activity, and stimuli responsiveness. Future progress in this field will depend on improved control over polyphenol stability, formulation reproducibility, and predictive gel design, thereby advancing next-generation silk-based gels and hydrogels for diverse functional applications.

## Figures and Tables

**Table 1:** SF–polyphenol gels: quantitative and semi-quantitative correlations between polyphenol structure and gel properties.

Polyphenol/Representative Structure	MW/Da	n(Galloyl)	n (Catechol/Pyrogallol-Type Motif)	Gelation Behavior	G′ or Stiffness Tendency	Dominant Interaction	Structure–Property Design Rule	References
TA	~1701.2	~10	Galloyl-derived pyrogallol-rich motifs	Short gelation time, low gelation concentration, adhesiveness, shear-thinning behavior, and self-recovery	G′ ≈ 1288 Pa	Hydrogen bonding, hydrophobic interaction, π–π stacking	Increasing galloyl density promotes rapid SF gelation and strong wet adhesion	[43,44]
Pentagalloyl glucose/pentagalloylglucose (PGG)	~940.7	5	5 galloyl-associated pyrogallol units	NR	NR	Multivalent hydrogen bonding, hydrophobic binding	Higher galloyl valency may enhance multipoint SF bridging	[45,46]
Epigallocatechin gallate (EGCG)	~458.4	1 ester galloyl	1 pyrogallol B-ring	Optimized tyramine-substituted silk fibroin (SF-T) 70/SF-EGCG30 hydrogels showed rapid in situ gelation within <30 s	G′ ≈ 1000 Pa	Hydrogen bonding and oxidative coupling	Combined galloyl and pyrogallol motifs favor injectable antioxidant SF hydrogels	[47,48]
Epicatechin gallate (ECG)	~442.4	1 ester galloyl	1 catechol B-ring	NR	NR	Hydrogen bonding and catechol oxidation	Replacing pyrogallol with catechol may reduce SF crosslink density	[49]
Gallic acid (GA)	~170.1	1 free galloyl-like unit	1 pyrogallol-type aromatic ring	NR	NR	Local hydrogen bonding	Low molecular valency limits standalone SF gelation capability	[50]
Caffeic acid (CA)	~180.2	0	1 catechol	NR	NR	Quinone-mediated chemistry	Catechol motifs are more effective after grafting or oxidative activation	[51,52]
Procyanidin B2/oligomeric catechol motif	~578.5	0	2 catechol-rich flavanol units	NR	NR	π–π stacking and hydrogen bonding/π–π	Increasing catechol copy number may improve oxidative network reinforcement	[53]

In this table, only TA and EGCG are supported by direct SF-based hydrogel studies. For PGG, ECG, gallic acid, caffeic acid, and procyanidin B2, direct standardized SF hydrogel data remain limited or unavailable. Therefore, these entries should be interpreted as structure-based hypotheses, mechanistic analogs, or auxiliary design motifs rather than experimentally validated SF gel performance rules. Entries marked as NR indicate that direct SF gel data under standardized conditions were not found.

**Table 2:** Comparison of representative SF–polyphenol complexes.

Composite Name	Form	Elastic Modulus	Strength	Adhesion	Rheology	Particle Size	EE (%)	Polyphenol/Drug Content or LC/DL (%)	References
G-RS/T10	Film	0.94 MPa	~0.18 MPa	~190 kPa on steel; ~30 kPa on wood	Newtonian behavior; Viscosity ~4 mPa·s	Thickness ~20 nm; Lateral size 16 μm	N/A	Tannin feed ratio: 10 wt%	[27]
SF–TA gel	Hydrogel	NR	NR	~7.3 kPa (at 0.1 wt% TA) to ~16.2 kPa (at 0.7 wt% TA)	G’ = 1288 Pa, G’’ = 177.2 Pa	43 µm (0.1% TA) decreasing to 26 µm (0.7% TA)	N/A	TA content/feed concentration: 0.1–0.7%	[43]
SFO–TA–BGNF	Aerogel scaffold	285 kPa	NR	-	G’ = 2649 Pa, G’’ = 385 Pa	37.6 nm	N/A	TA content/feed ratio: 10%	[54]
m-SF/TA/Zn^2+^	Hydrogel	NR	NR	-	G’ > G’’	3622 nm	N/A	TA content/feed ratio: 1.33%	[55]
SF–TA	Hydrogel	0.3 ± 0.1 MPa	Toughness: 123.1 ± 11.5 kJ/m^3^	86.1 ± 6.4 kPa (within 1 min); 134.1 ± 5.2 kPa (reached in 20 min)	G’ > G’’	~30 nm	N/A	NR	[17]
SC_15_(TW)_5_	Hydrogel	1.6 kPa–180 MPa	Toughness: ~40 kJ/m^2^	~70 kPa	NR	20–30 nm	N/A	NR	[56]
SFMA–TP	Hydrogel	6.42–24.35 kPa	Compressive strength: 8.5–32.5 kPa; Burst Pressure: 126 ± 9 mmHg	28–32 kPa	G’ = 200–3500 Pa	40–100 μm	N/A	TP content/feed ratio: 40–60%	[57]
TP-DA/SF	Hydrogel	NR	NR	Porcine skin: 95.21 kPa; Wood: 110 kPa; Steel: 93 kPa; Glass: 85 kPa; PTFE: 26 kPa	G’ > G’’	NR	N/A	NR	[26]
SFO-SSDopa-Cu-TA	Hydrogel	1.72 ± 1.25 kPa	Stress: 4.73 ± 1.43 kPa Toughness: 0.982 ± 0.34 kJ/m^3^	Bone-mimicking substrate: 854.15 ± 12.90 kPa Tissue-mimicking substrate: 664.03 ± 15.87 kPa	Self-healing recovery: 72.27 ± 9.35%; Yield stress: 126.14 ± 6.57 Pa; G’ = 7025 ± 1709 Pa; G’’ = 849 ± 240 Pa	NR	N/A	NR	[25]
EGCG–SF–MNs	Microneedle patch	NR	NR	NR	NR	Needle height: ~790 μm Base width: ~297 μm Tip-to-tip spacing: ~700 μm	NR	EGCG loading/content: 16.7%	[58]
NAR–SFNs	Nanoparticles	NR	NR	NR	NR	Loading ratio (1:4): 157.6 ± 0.9 nm Loading ratio (1:2): 164.0 ± 1.1 nm Loading ratio (1:1): 180.1 ± 2.6 nm	Loading ratio (1:4): 17.33 ± 0.70% Loading ratio (1:2): 19.50 ± 0.45% Loading ratio (1:1): 21.81 ± 0.30%	Loading ratio (1:4): 4.18 ± 0.19% Loading ratio (1:2): 7.62 ± 0.20% Loading ratio (1:1): 21.82 ± 0.40%	[59]
SF-BGE/TA	Coacervates	40.2 ± 3.5 kPa	NR	Dry wood: 0.44 ± 0.05 MPa Wood (90% humidity): 0.77 ± 0.08 MPa Underwater (pH 6): 1.07 ± 0.12 MPa Underwater (pH 3): 0.21 ± 0.03 MPa	G’ > G’’	NR	N/A	TA content/feed ratio: 20%	[60]

G-RS/T10: Graphene-regenerated silk/tannin (10%); SF–TA: Silk fibroin–tannic acid; G’: Storage modulus; G’’: Loss modulus; SFO–TA–BGNF: Oxidized silk fibroin–tannic acid–bioactive glass nanofibers; m-SF/TA/Zn^2+^: Water-soluble silk fibroin/tannic acid/Zinc ion; SC_15_(TW)_5_: silk fibroin/CaCl_2_/tannic acid−tungsten disulfide 2; SFMA–TP: methacrylated silk fibroin–tea polyphenols; TP-DA/SF: Tea polyphenols-dopamine/silk fibroin; SFO-SSDopa-Cu^2+^-TA: Oxidized silk fibroin/dopamine-modified silk sericin/copper ions/tannic acid hybrid hydrogel; EGCG–SF–MNs: Epigallocatechin gallate-loaded silk fibroin microneedles; NAR–SFNs: Naringenin-loaded silk fibroin nanoparticles; SF-BGE/TA: silk fibroin-butyl glycidyl ether/tannic acid; EE (%): Encapsulation efficiency; LC/DL (%): Loading content/drug loading. For systems in which polyphenols serve primarily as network-forming, adhesive, crosslinking, antioxidant, coordination, or reinforcing components rather than encapsulated cargo, EE was considered not applicable and marked as “N/A”. When the original literature did not report the corresponding value, the entry was marked as “NR”. When only the feed ratio or formulation content of polyphenols was available, it was reported as polyphenol/drug content rather than true drug-loading capacity.

**Table 3:** Key figures of merit for comparing representative SF–polyphenol gels and hydrogel-derived materials.

Figure of Merit	Representative Reported Range or Value	Mechanistic Interpretation	References
Gelation time	<30 s (SF-EGCG), ~11 h (0.1 wt% TA), 9.5–13.8 min (SF–TA + HRP/H_2_O_2_)	Fast gelation relies on high-valency galloyl-mediated physical crosslinking or enzymatic oxidation; slow gelation indicates weak physical assembly or low crosslink density.	[42,43,47]
Storage modulus (G′)	1000 Pa (SF-EGCG), 1288 Pa (SF–TA), 7025 Pa (Cu/TA hybrid)	Storage modulus increases with stronger physical interactions and additional covalent or coordination crosslinks; excessively high stiffness may affect material flexibility.	[25,43,47]
Wet adhesion	7.3–16.2 kPa (SF–TA), 150–180 kPa (TASK adhesive), >600 kPa (Cu/TA hybrid)	Adhesion arises from interfacial phenolic groups, dynamic coordination, and internal hydrogel network structure; water and substrate type influence final adhesive performance.	[18,25,43]
Antioxidant/ROS response	DPPH scavenging 76–97%, intracellular ROS inhibition up to 95.5%	Antioxidant performance depends on the preservation of phenolic hydroxyl groups; excessive oxidation or radical curing can consume active phenols.	[42,90]
Electrical/strain sensing	Gauge factor 0.11 → 1.16 (0 → 20 wt% MXene)	Conductive fillers form the main electrical pathway; TA protects phenolic groups from oxidation, helping maintain long-term signal stability.	[91]

**Table 4:** pKa-dependent ionization behavior and recommended pH ranges for silk fibroin–polyphenol solution blending and in situ assembly.

Polyphenol	pKa	Ionization Characteristics	Recommended pH Range	Stabilization Rationale	References
TA	pKa1 ≈ 5.6; pKa2 ≈ 6.9; pKa3 ≈ 8.1	Stepwise dissociation of multiple phenolic hydroxyls; negative charge increases and H-bond donating ability decreases at neutral to mildly alkaline pH.	pH 3.0–6.5	Mildly acidic pH keeps most phenolic hydroxyls protonated, favoring multivalent H-bonding, hydrophobic/π–π interactions, and reduces high-pH oxidation and aggregation.	[43,98,100]
EGCG	pKa ≈ 7.5–7.7	Gradual deprotonation; high pH promotes autoxidation, epimerization, dimerization.	pH 4.0–6.5	Mildly acidic pH minimizes autoxidation and dimer formation, allowing H-bonding and grafting interactions with SF; preserves antioxidant activity.	[47,101,103]
GA	Carboxyl pKa ≈ 4.4–4.7; phenolic pKa values are generally >8.5	Carboxyl dissociates first, phenolic hydroxyls at higher pH.	pH 3.0–5.0	Partial carboxyl ionization improves solubility; protonated phenolics reduce oxidation and coupling reactions.	[99,104]
Methyl gallate (MeG)	pKa1 = 7.96 ± 0.01; pKa2 = 10.97 ± 0.02	C4′–OH deprotonates first; further deprotonation possible at C3′/C5′.	pH 2.0–7.0	Low to near-neutral pH limits quinonoid formation and oxidative reactions.	[105]
CA	pKa1 ≈ 4.02; pKa2 ≈ 8.43	Carboxyl dissociates first; catechol hydroxyls deprotonate at high pH.	pH 2.0–6.0	Acidic conditions maintain solubility and limit excessive phenolic deprotonation, promoting homogeneous dispersion.	[99,106,107]
Quercetin	pKa1 ≈ 8.45; pKa2 ≈ 9.3; pKa3 ≈ 11.1	7–OH preferentially deprotonates; deprotonation increases reactivity.	pH 3.0–7.0	Protonated phenolic groups favor multivalent H-bonding, π–π interactions, and antioxidant stability.	[108,109,110]
Ferulic acid (FA)	Carboxyl pKa ≈ 4.3–4.6; phenolic pKa ≈ 8.6–8.8	Carboxyl dissociates first; phenolic hydroxyl deprotonates at high pH.	pH 3.0–6.5	Mildly acidic pH balances solubility and oxidative stability of phenolic groups.	[99,107]
Rosmarinic acid (RA)	Carboxyl pKa ≈ 3.6;	Carboxyl dissociates first; catechol moieties deprotonate under neutral to mildly alkaline conditions.	pH 3.0–7.0	Mildly acidic to near-neutral pH limits catechol oxidation while maintaining solubility; avoids overly acidic/alkaline conditions.	[111,112]

GA: Gallic acid; MeG: Methyl gallate; CA: Caffeic acid; FA: Ferulic acid; RA: Rosmarinic acid.

**Table 5:** Overview of fabrication methods, characteristics, and applications for SF–polyphenol complexes.

Fabrication Strategy	Interaction Type	Key Advantages	Limitations	Typical Applications	References
Solution blending	Physical (H–bonds, hydrophobic)	Mild conditions; Preserves polyphenol bioactivity; Simple and scalable	Weaker mechanical strength; Susceptible to swelling/disintegration in water	Injectable hydrogels; Coatings	[18,20,37,67,102,132]
Electrospinning	Physical/Electrostatic	High specific surface area; Tunable porosity; Mimics ECM structure	Requires organic solvents (often); Complex equipment; Lower yield	Wound dressings; Tissue scaffolds	[36,66,113,114,115]
Impregnation–adsorption	Physical Adsorption	Versatile post-functionalization; Retains original substrate architecture	Risk of burst release; Cargo leakage due to reversible binding	Drug delivery; Surface functionalization	[19,56,59,116,117]
Enzymatic crosslinking	Covalent (C–N, C–O bonds)	High physiological stability; Tunable degradation; Dense network	Costly enzymes; Reaction requires strict pH/temperature control	Tissue adhesives; Long-term implants	[32,35,119]
Metal–phenolic coordination	Coordination (Fe^3+^, Zn^2+^)	Self-healing properties; Stimuli-responsive (pH); Wet adhesion	Potential cytotoxicity of metal ions; Risk of discoloration	Bio-adhesives; Smart coatings	[18,20,25,120,121]
Photopolymerization	Covalent (Radical)	Spatiotemporal control; Rapid gelation; 3D printable	Polyphenols act as radical scavengers (slowing curing); Potential ultraviolet (UV) damage	3D printing inks; Bioelectronics	[20,122,123,124,125,126,131,133]

SF: Silk fibroin; ECM: extracellular matrix; UV: ultraviolet.

**Table 6:** Quantitative comparison of representative stimulus-responsive silk fibroin–polyphenol systems.

Stimulus Type	Stimulus Condition	Quantitative Results	Reference
TA-induced gelation/physiological-condition response	1.0% SF; TA = 0.1–0.7 wt%; pH 7.4; 37 °C	Pure SF: no gel in 30 d. +0.1 wt% TA: gelation ~11 h. Higher TA → shorter gelation time.	[43]
Metal–polyphenol coordination/antioxidant and antibacterial response	m-SF/TA with ZnCl_2_; TA: ZnCl_2_ = 2:5; stirred 12 h at room temperature	Antioxidant activity: m-SF 23.5%, m-SF/TA solution 79.7%, hydrogel 79.3%. Inhibition zones: *E. coli* 7.9 mm, *S. aureus* 8.1 mm.	[55]
Wet-adhesion response	TASK powder mixed with water at ~1:1 mass ratio to form composite gel	Adhesion strength reached 150.3 kPa after 3 h and 180.9 kPa after 5 h in water; ~10 kPa on tissues.	[18]
NIR photothermal response/photothermal-enhanced antibacterial activity	SF, TA, and Fe_3_O_4_ NPs; 808 nm NIR irradiation at 0.25 W cm^−2^	Temperature: High Fe_3_O_4_ groups > 50 °C within 3 min. Bactericidal ratio (without NIR): STFe-0 → ~77% (*S. aureus*), ~56% (*E. coli*). With NIR: STFe-0.1 & STFe-0.2 → >93% (*S. aureus*), >94% (*E. coli*).	[20]
Reactive oxygen species (ROS)/oxidative-stress response	EGCG-grafted SF + Tyramine-SF; HRP/H_2_O_2_ crosslink	Gelation < 30 s, storage modulus ~1000 Pa. Superior healing vs. DuoDERM^®^ in rat model.	[47]
ROS/osteochondral-defect oxidative microenvironment response	4% SF + 0.005% TA; HRP/H_2_O_2_; rabbit model	Gelation time: 13.8 min (25 °C), 9.5 min (37 °C). Compressive modulus 29.2 kPa. DPPH scavenging: SF-TA 76.3%, SF-TA-E7 67.6%. Intracellular ROS inhibition: 89.9% (SF-TA), 95.5% (SF-TA-E7). BMSC viability under H_2_O_2_: 75.2% (SF), 85.0% (SF-TA-E7). E7 release at day 21: 63.9% (SF-E7) → 43.8% (SF-TA-E7).	[42]
ROS/anti-inflammatory/bone-regeneration response	FMA-LAP + TA 5–15 mg/mL; UV crosslink	DPPH scavenging up to 96.7% (TA 15 mg/mL). Reduced IL-6/TNF-α, increased COL-I, Runx-2, OCN, OPN. Best BV/TV & BMD in 8-week cranial defect.	[90]
Electrical/strain response and MXene oxidation protection	TA-crosslinked SF/MXene hydrogel; MXene 0–20 wt%	Gauge factor: 0.11 (0% MXene) → 1.16 (20% MXene). Electrical recovery after cutting ~0.5 s. TiO_2_ formation after H_2_O_2_: 53.2% (without TA) vs. 7.8% (with TA). Stable signal for 10 d at RT.	[91]

NIR: near-infrared; ROS: Reactive oxygen species; TASK: TA–SF composite adhesive; COL-I: collagen type I; Runx-2: runt-related transcription factor 2; OCN: osteocalcin; OPN: osteopontin; BV/TV: bone volume/total volume; BMD: bone mineral density; LAP: laponite; BMSC: bone marrow mesenchymal stem cells; m-SF/TA: water-soluble silk fibroin solution; STFe: silk fibroin/tannic acid/iron oxide nanoparticle; DPPH: 2,2-Diphenyl-1-picrylhydrazyl; MXene: carbonitride nanosheet; TiO_2_: Titanium dioxide.

## Data Availability

No new data were created or analyzed in this study. Data sharing is not applicable to this article.

## Abbreviations

The following abbreviations are used in this manuscript:

^13^C CP-MAS NMR	Carbon-13 Cross-Polarization Magic-Angle Spinning Nuclear Magnetic Resonance
AD	Adamantane
ATR-FTIR	Attenuated Total Reflectance Fourier Transform Infrared Spectroscopy
AFM	Atomic force microscopy
BMD	Bone mineral density
BMSCs	Bone marrow mesenchymal stem cells
BV/TV	Bone volume/Total volume
CD	Circular dichroism
COL-I	Collagen type I
Cur	Curcumin
CuTA	Copper–tannic acid
DLS	Dynamic light scattering
DL	Drug loading
DPPH	2,2-Diphenyl-1-picrylhydrazyl
DSC	Differential scanning calorimetry
ECG	Electrocardiography
ECM	Extracellular matrix
*E. coli*	*Escherichia coli*
EDOT	3,4-Ethylenedioxythiophene
EE	Encapsulation efficiency
EGCG	Epigallocatechin gallate
EGCG–SF–MNs	Epigallocatechin gallate-loaded silk fibroin microneedles
EMG EtO FDA	Electromyography Ethylene oxide Food and Drug Administration
FTIR	Fourier transform infrared spectroscopy
G’	Storage modulus
G’’	Loss modulus
GA	Gallic acid
GR	Graphene
G-RS/T10	Graphene-regenerated silk/tannin (10%)
GuCl	Guanidinium chloride
H_2_O_2_	Hydrogen peroxide
H-chains	Heavy chains
HO-1	Heme oxygenase-1
HRP	Horseradish peroxidase
LAP	Laponite
IL-1β	Interleukin-1 beta
L-chains	Light chains
LC	Loading content
LiBr	Lithium bromide
MOFs	Metal–organic frameworks
MPNs	Metal–phenol networks
MRSA	Methicillin-resistant **Staphylococcus aureus**
m-SF	Water-soluble silk fibroin
m-SF/TA	Water-soluble silk fibroin/tannic acid
m-SF/TA/Zn^2+^	Water-soluble silk fibroin/tannic acid/zinc ion
MXene/SF/TA	MXene/silk fibroin/tannic acid
NAR–SFNs	Naringenin-loaded silk fibroin nanoparticles
NIR	Near-infrared
NO	Nitric oxide
Nrf2	Nuclear factor erythroid 2-related factor 2
OCN OAChN	Osteocalcin Coassembly of multi-surface-charged chitin nanofibers
OPN	Osteopontin
PBS	Phosphate-buffered saline
PDA	Polydopamine
PDA-Fe PEDOT/SF	Polydopamine–iron/poly(3,4-ethylenedioxythiophene)/silk fibroin
PEDOT	Poly(3,4-ethylenedioxythiophene)
pH	Potential of hydrogen
PPy	Polypyrrole
PVA	Polyvinyl alcohol
PVA/SF/TA/GR	Polyvinyl alcohol/silk fibroin/tannic acid/graphene
PVA borax SF/TA	Polyvinyl alcohol/borax/silk fibroin/tannic acid
PBST	Polyvinyl alcohol/borax/silk fibroin/tannic acid system
QSARs	Quantitative structure–activity relationships
ROS	Reactive oxygen species
RS	Regenerated silk
RSF	Regenerated silk fibroin
Runx-2	Runt-related transcription factor 2
SC15(TW)5	Silk fibroin/CaCl2/tannic acid–tungsten disulfide
SEM	Scanning electron microscopy
SF	Silk fibroin
SF/Gel/GA	Silk fibroin/gelatin/gallic acid
SF–TA	Silk fibroin–tannic acid
SF/TA/Fe3+	Silk fibroin/tannic acid/iron (III)
SF/TA/PPy	Silk fibroin/tannic acid/polypyrrole
SF-BGE/TA	Silk fibroin/butyl glycidyl ether/tannic acid
SFMA	Methacrylated silk fibroin
SFMA–TPs	Methacrylated silk fibroin–tea polyphenols
SFNs	Silk fibroin nanoparticles
SFO–TA–BGNF	Oxidized silk fibroin/tannic acid/bioactive glass nanofibers
SFO-SSDopa-Cu-TA	Oxidized silk fibroin/dopamine-modified silk sericin/copper ions/tannic acid
SOD-1	Superoxide dismutase-1
*S. aureus*	*Staphylococcus aureus*
STIG	Silk fibroin/tannic acid/ibuprofen/guanidinium chloride hydrogel
STFe	Silk fibroin/tannic acid/iron oxide nanoparticle bioadhesive
TA	Tannic acid
TASK	TA–SF composite adhesive
SF-T	Tyramine-substituted silk fibroin
TEM	Transmission electron microscopy
TiO_2_	Titanium dioxide
TME	Tumor microenvironment
TPs	Tea polyphenols
TP-DA/SF	Tea polyphenols-dopamine/silk fibroin
UV	Ultraviolet
UV-Vis	Ultraviolet-visible spectroscopy
XRD	X-ray diffraction
Zn^2+^	Zinc ion
